# Transcriptomic Signatures and Functional Network Analysis of Chronic Rhinosinusitis With Nasal Polyps

**DOI:** 10.3389/fgene.2021.609754

**Published:** 2021-02-02

**Authors:** Yun Hao, Yan Zhao, Ping Wang, Kun Du, Ying Li, Zhen Yang, Xiangdong Wang, Luo Zhang

**Affiliations:** ^1^Department of Otolaryngology Head and Neck Surgery, Beijing TongRen Hospital, Capital Medical University, Beijing, China; ^2^Department of Allergy, Beijing TongRen Hospital, Capital Medical University, Beijing, China; ^3^Beijing Key Laboratory of Nasal Diseases, Beijing Institute of Otolaryngology, Beijing, China; ^4^Shanghai Key Laboratory of Medical Epigenetics, The International Co-laboratory of Medical Epigenetics and Metabolism, Ministry of Science and Technology, Pudong Hospital, Institutes of Biomedical Sciences, Fudan University, Shanghai, China; ^5^Peking Union Medical College, Chinese Academy of Medical Sciences, Beijing, China

**Keywords:** chronic rhinosinusitis with nasal polyps, differentially expressed genes, hub genes, transcriptomic functional features, drug repurposing bioinformatics analysis of nasal polyps

## Abstract

Chronic rhinosinusitis with nasal polyps (CRSwNP) is a chronic sinonasal inflammatory disease with limited treatment options of corticosteroids, sinus surgery, or both. CRSwNP is frequently associated with allergic rhinitis and asthma, but the molecular mechanisms underlying CRSwNP inflammation are not completely understood. We obtained four gene expression profiles (GSE136825, GSE36830, GSE23552, and GSE72713) from four Gene Expression Omnibus (GEO), which collectively included 65 nasal polyp samples from CRSwNP patients and 54 nasal mucosal samples from healthy controls. Using an integrated analysis approach, we identified 76 co-differentially expressed genes (co-DEGs, including 45 upregulated and 31 downregulated) in CRSwNP patients compared with the healthy controls. Gene Ontology (GO) and Kyoto Encyclopedia of Genes and Genomes (KEGG) analyses identified the terms including immune effector process, leukocyte migration, regulation of the inflammatory response, *Staphylococcus aureus* infection, and cytokine-cytokine receptor interaction. protein-protein interaction (PPI) network analysis and real-time quantitative PCR (RT-qPCR) showed that 7 genes might be crucial in CRSwNP pathogenesis. Repurposing drug candidates (Alfadolone, Hydralazine, SC-560, Iopamidol, Iloprost, etc) for CRSwNP treatment were identified from the Connectivity Map (CMap) database. Our results suggest multiple molecular mechanisms, diagnostic biomarkers, potential therapeutic targets, and new repurposing drug candidates for CRSwNP treatment.

## Introduction

Chronic rhinosinusitis (CRS) is a common chronic heterogeneous nasal inflammatory disease that is associated with significant morbidity and a decreased quality of life. It affects ~7 to 27% of adults in European populations, 14% of adults in the United States, and 8% of adults in China (Hastan et al., 2011; Shi et al., 2015; Wang X. et al., 2016). CRS is clinically classified into two phenotypes according to the presence or absence of nasal polyps: CRS with nasal polyps (CRSwNP) and CRS without nasal polyps (CRSsNP) (Workman et al., 2018). CRSwNP can be classified into 2 distinct immunohistological subtypes based on eosinophil infiltration, eosinophilic CRSwNP (Eos CRSwNP) and non-eosinophilic CRSwNP (non-eos CRSwNP) (Cao et al., 2009). Eos CRSwNP demonstrates Th2 inflammation skewed with a relatively high recurrence and asthma comorbidity rate, while non-eos CRSwNP is characterized by a Th1 or Th17 response and a lower recurrence and asthma comorbidity rate (Zhang et al., 2008; Cao et al., 2009).

Recent studies have demonstrated that defects in the sinonasal epithelial barrier, increased exposure to pathogenic and colonized bacteria, and dysregulation of the host immune system play key roles in CRSwNP pathogenesis (Stevens et al., 2016). However, the inflammatory mechanisms underlying CRSwNP are not completely defined. In this regard, biomarkers that precisely indicate the development and progression of CRSwNP need to be further investigated to develop novel clinical strategies for CRSwNP treatment.

Microarray technology and bioinformatic analysis have emerged as promising, useful tools for screening genetic alterations involved in the development and progression of diseases. Furthermore, over the last decade, next-generation sequencing has produced substantial improvements in quality and yield (Goodwin et al., 2016). However, obtaining reliable results is difficult with both individual microarrays and sequencing due to the lack of samples (Kulasingam and Diamandis, 2008). Therefore, to obtain further insights into the mechanisms underlying the pathogenesis of CRSwNP and to clarify potential therapeutic targets, we analyzed a sufficient number of samples and combined differentially expressed genes (DEGs) derived from multiple microarray datasets with sequence-based data.

We herein aimed to explore the possible molecular mechanisms and biomarkers and propose new drug candidates for CRSwNP by integrating all the public databases for CRSwNP and using bioinformatics analyses of co-differentially expressed genes (co-DEGs) in nasal polyps from CRSwNP patients compared to nasal mucosal tissues from healthy control tissues. We described the transcriptional features, identified the biomarkers, and predicted the drug repurposing candidates, which could provide insights into precise CRSwNP treatment strategies.

## Materials and Methods

### Microarray Studies, Datasets and Characteristics of Clinical Samples From the GEO Data Repository

**Figure 1:**
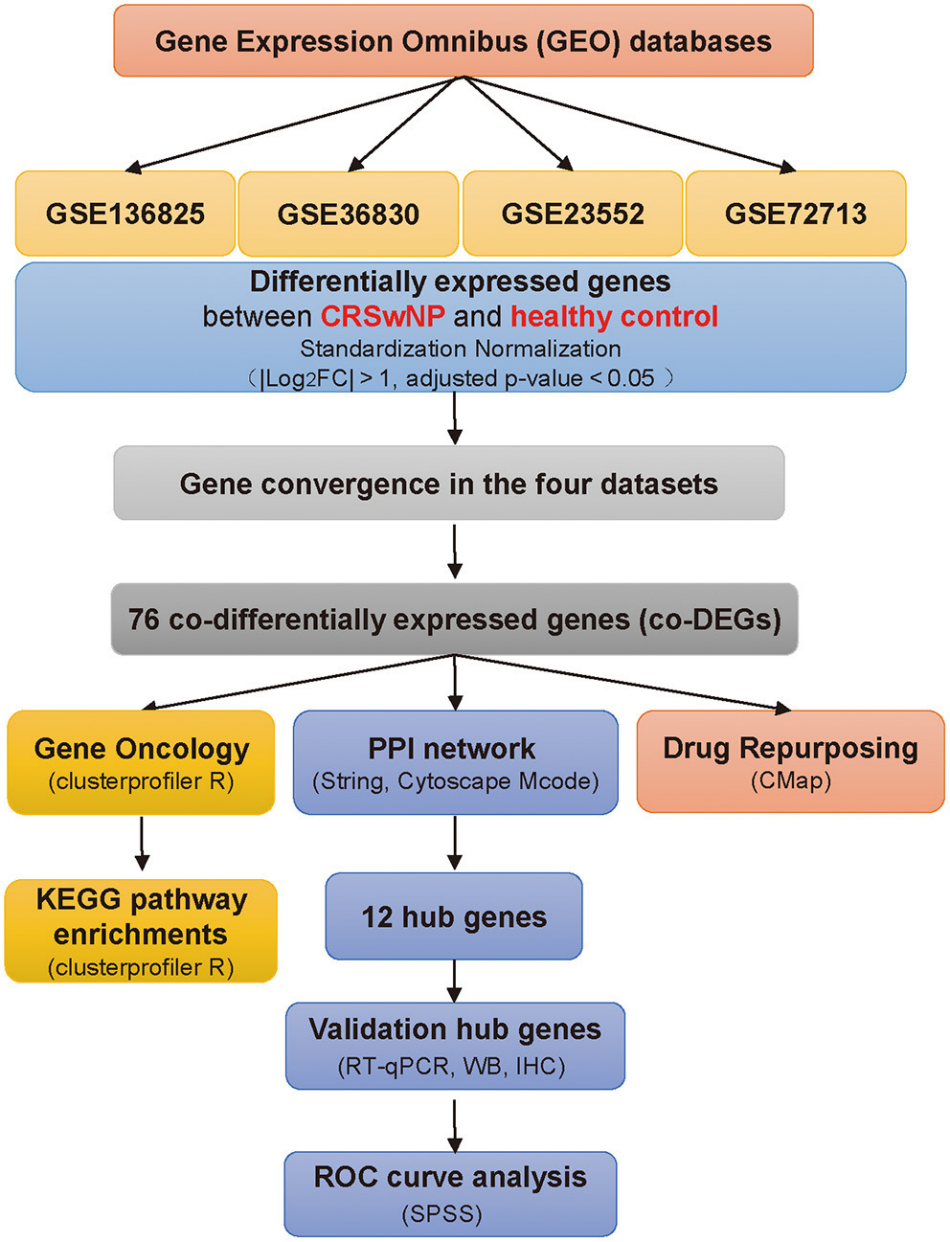
Schematic overview of the study. Flowchart of the study to identify potential factors underlying CRSwNP pathogenesis compared with healthy controls. CRSwNP, chronic rhinosinusitis with nasal polyps; GO, Gene Ontology; FC, fold change; Gene Ontology; KEGG, Kyoto Encyclopedia of Genes and Genomes; PPI, protein-protein interaction; co-DEGs, co-differentially expressed genes; CMap, Connectivity Map database; RT-qPCR, real-time quantitative PCR; WB, western blot; IHC, immunohistochemistry.

In the present study, we selected microarray and high-throughput sequencing datasets of nasal tissues from CRSwNP patients in the GEO database using the following keywords: “CRSwNP,” “Homo sapiens,” and “nasal tissue.” Based on these keywords, four CRSwNP datasets (GSE136825, GSE36830, GSE23552, and GSE72713) were downloaded from the repository. Derived from the GPL20301 platform (Illumina HiSeq 4000), GSE136825 includes nasal polyp tissue samples from 42 CRSwNP patients and nasal mucosal samples from 28 healthy controls (Peng et al., 2019). GSE36830 includes nasal polyp tissue samples from 6 CRSwNP patients and nasal mucosal samples from 6 healthy controls evaluated with the GPL570 platform (Affymetrix Human Genome U133 Plus 2.0 Array) (Stevens et al., 2015b). GSE23552 is based on the Affymetrix Human Exon 1.0 ST Array and includes nasal polyp tissue samples from 11 CRSwNP patients and nasal mucosal samples from 17 healthy controls (Plager et al., 2010). GSE72713 is based on an Illumina HiSeq 2000 and includes nasal polyp tissue samples from 6 CRSwNP patients and nasal mucosal samples from 3 healthy controls (Wang W. et al., 2016). The details of each dataset are shown in Table 1 and Supplementary Table 10. The flow chart detailing this study protocol is shown in Figure 1.

**Table 1:** The details of GEO datasets for CRSwNP.

**GSE**	**PMID**	**Sample size (*n*)**	**Technology**	**Platform**	**Instrument**	**Age (y)**	**Sex, male (*n*%)**	**Number of DEGs**	**mRNA**	
									**Up**	**Down**
GSE136825	PMID: 31439685	CRSwNP: 42 Control: 28	High-Throughput sequencing	GPL20301	Illumina HiSeq 4000	NA	NA	851	507	344
GSE36830	PMID: 26067893	CRSwNP: 6 Control: 6	*In situ* oligonucleotide	GPL570	[HG-U133_Plus_2] Affymetrix Human Genome U133 Plus 2.0 Array	CRSwNP: 38 ± 5 Control: 36 ± 6	CRSwNP: 4 (67%) Control: 2 (33%)	286	149	137
GSE23522	PMID: 20625511	CRSwNP: 11 Control: 17	*In situ* oligonucleotide	GPL5175	[HuEx-1_0-st] Affymetrix Human Exon 1.0 ST Array [transcript (gene) version]	CRSwNP: 40 ± 2.788 Control: 31.22 ± 2.913	CRSwNP: 5 (45%) Control: 9 (53%)	459	271	188
GSE72713	PMID: 27216292	CRSwNP: 6 Control: 3	High-Throughput sequencing	GPL11154	Illumina HiSeq 2000 (Homo sapiens)	CRSwNP: 46.8 ± 4.8 Control: 48.7 ± 7.6	CRSwNP: 4 (67%) Control: 1 (67%)	85	21	64

### Differential Gene Expression Analysis

First, background correction and standardization were performed for the original GEO datasets using the packages EdgeR and Limma of R software (Ritchie et al., 2015). To determine whether the DEGs distinguished the CRSwNP group from healthy controls, principal coordinate analysis (PCoA) was applied to compare the overall characteristics of DEG communities between the two groups. The PCoA results were extracted and visualized using the Vegan and Ggplot2 packages of R software (version 1.2.5033) (Zhang et al., 2019). Next, differential analysis (|log_2_FC| > 1, adjusted *p* < 0.05) of mRNAs was performed to compare nasal polyp and normal tissue samples with the Limma package of R software. Heatmaps and volcano plots of differentially expressed mRNAs were constructed using the packages Pheatmap and Ggplot2 of R software.

Subsequently, a Venn diagram showing the intersecting DEGs of the four datasets was created with Funrich software (version 3.1.3) (Pathan et al., 2015). The raw data in the four datasets are summarized in the form of a matrix and are shown in Supplementary Table 1.

### PPI Network Construction

STRING (version 11.0) (http://string-db.org/) was used to identify the PPIs of the intersecting DEGs of the four datasets, with a combined score >0.4 used as the threshold for statistically significant interactions(Szklarczyk et al., 2015). Cytoscape (version 3.7.2) software was used for the PPI network visualization (Shannon et al., 2003). Then, the Molecular Complex Detection (MCODE) plugin, a graph theoretic clustering algorithm finding highly interconnected regions in a given network was used to identify important modules within the PPI network (Bader and Hogue, 2003). For this algorithm, seed vertices are selected and expanded by the density of the cluster. In detail, the degree cutoff was 2, the node score was 0.2, the k-score was 2, and the maximum depth was 100. A false discovery rate (FDR) < 0.05 was considered statistically significant.

### Functional Enrichment and Pathway Analyses

GO functional enrichment analysis is a commonly used method for annotating genes and identifying characteristic biological attributes of high-throughput genome or transcriptome data (Ashburner et al., 2000; Gene Ontology, 2006). KEGG pathway analysis is well-known for its systematic analysis of gene functions in biological pathways, which links genomic information with higher-order functional information (Kanehisa and Goto, 2000). Clusterprofiler package of R software integrates GO functional enrichment and KEGG pathway analyses (Yu et al., 2012). We analyzed the functions and signaling pathways of the intersecting DEGs using GO and KEGG analyses by ClusterProfiler package of R software. GO annotation includes three kinds of functional categories: biological process (BP), cellular component (CC) and molecular function (MF). *P* < 0.05 and *q* < 0.2 were considered statistically significant.

### Screening Candidate Small-Molecule Drugs

To screen potential small-molecule drugs related to CRSwNP, the Connectivity Map (CMap) database, an online program for predicting potential drugs that may affect the biological status encoded by specific gene expression markers (https://portals.broadinstitute.org/cmap/), was employed (Lamb et al., 2006). Co-DEGs, which included upregulated and downregulated genes, were uploaded to query the CMap database. The enrichment score indicative of similarity was calculated and ranged from −1 to 1. A positive connectivity score indicated that the drug could induce the expression of the queried gene in CRSwNP, while a negative connectivity score indicated that the drug induced a status similar to that of normal cells, suggesting its potential to treat CRSwNP. The results were ranked by *p*-value.

### Patient Recruitment

This study was approved by the Ethics Committee of Beijing TongRen Hospital, Capital Medical University, and written informed consent was obtained from each patient before enrollment. A total of 70 subjects, including 46 patients with CRSwNP and 24 healthy control subjects, were recruited. We collected nasal polyp tissues from patients with CRSwNP and nasal mucosal tissues from control subjects. The diagnosis of CRSwNP was made according to the European Position Paper on Rhinosinusitis and Nasal Polyps 2012 guidelines (Fokkens et al., 2012). Control subjects without other sinonasal diseases were those undergoing septoplasty because of anatomic variations. None of the patients had been treated with corticosteroids, immunomodulatory agents, or antibiotics within 4 weeks before enrollment. The exclusion criteria were as follows: patients with acute infections, acetylsalicylic acid-intolerance, fungal sinusitis, immunodeficiency, coagulation disorder, or cystic fibrosis and pregnant women. Details of the subjects' characteristics are included in Supplementary Table 7.

### RNA Extraction and Real-Time Quantitative PCR (RT-qPCR)

Total RNA was isolated from nasal polyps of CRSwNP patients and from the nasal mucosa of controls using Tri®-Reagent (Sigma) according to the manufacturer's instructions. The quality of total RNA was assessed with a Nanodrop-2000 (Thermo Fisher Scientific, Waltham, Mass), and complementary DNA was synthesized from 1 μg of total RNA using PrimeScript RT Master Mix (Abclonal Biotechnology). RT-qPCR was performed by using SYBR Green mix (Abclonal Biotechnology) to assess gene expression levels. Primers are listed in Supplementary Table 8.

### Western Blot Analysis

Tissues of nasal polyps from CRSwNP patients and the nasal mucosa of controls were homogenized in ice-cold RIPA lysis buffer (50 mM Tris-HCl, pH 7.5, 150 mM NaCl, 1.0% Triton X-100, 20 mM EDTA, 1 mM Na_3_VO_4_, 1 mM NaF, and 1 mM PMSF). The protein concentration was measured by bicinchoninic acid (BCA) kit (Beyotime, Shanghai, China). In brief, equal amounts of proteins (16 μg) were loaded on the sodium dodecyl sulfate–polyacrylamide gel electrophoresis (SDS–PAGE) and transferred to nitro cellulose membranes. The membranes were sequentially incubated with primary antibodies and horseradish peroxidase-conjugated secondary antibodies (described below). The following primary antibodies were used: anti-BTK (1:1000 diluted, ABclonal, A19002, Wuhan, China), anti-HCK (1:1000 diluted, ABclonal, A14537, Wuhan, China), anti-HK3 (1:1000 diluted, ABclonal, A8428, Wuhan, China), anti-NCF2 (1:1000 diluted, ABclonal, A1178, Wuhan, China), anti-NOX2/gp91phox (1:1000 diluted, Abcam, ab80897), anti-FLAP (3:5000 diluted, Abcam, ab53536), and anti-β-actin (1:10000 diluted, Sigma, A5441) at 4°C overnight, they were further immunoblotted with HRP-conjugated IgG antibody (1:5000 diluted, ABclonal, Wuhan, China) at room temperature for 60 min, developed with enhanced chemiluminescence (ECL) substrate (Millipore, Darmstadt, Germany) and chemiluminescence detection by ChemiDocTM MP Imaging System (Bio-Rad, United Kingdom). Band density was quantitated using the Image LabTM software Version 6.0.0 (Bio-Rad, United Kingdom).

### Immunohistochemistry Staining

Five-micron thick sections were obtained from blocks of nasal polyps and nasal mucosa from CRSwNP patients and control subjects, dewaxed in xylol and rehydrated in graded ethanol. For antigen retrieval, the slides containing the samples were incubated with citrate buffer (pH 6.0) in a pressure cooker (Zhongshan Jinqiao Biotechnology, Beijing, China). The samples were then treated with freshly prepared 3% hydrogen peroxide in methanol for 20 min and further washed in Tris-buffered saline. The slides were incubated overnight at 4°C with anti-BTK (1:100 diluted, ABclonal, A19002, Wuhan, China), anti-HCK (1:200 diluted, ABclonal, A2083, Wuhan, China), anti-HK3 (1:400 diluted, ABclonal, A8428, Wuhan, China), anti-NCF2 (1:500 diluted, ABclonal, A1178, Wuhan, China), anti-NOX2/gp91phox (1:100 diluted, ABclonal, A19701, Wuhan, China), anti-BFL-1/GRS (1:150 diluted, Abcam, ab45413), anti-FLAP (1:100 diluted, Abcam, ab53536). A polymer system (Zhongshan Jinqiao Biotechnology, Beijing, China) was applied as a secondary antibody conjugated to peroxidase. DAB (3′-diaminobenzidine tetrahydrochloride, Zhongshan Jinqiao Biotechnology, Beijing, China) was used as the chromogen, for 5 min, followed by Harris hematoxylin counterstain. Slides were analyzed under a light microscope (Nikon H600L, Japan) and 5 images were taken for each slide (Nikon NIS software, version 4.60, Nikon, Japan) at high-power (40X objective) field. Representative areas were qualitatively selected for immunostaining analysis. For digital analysis, we used the cell counter function of the ImageJ software (version 1.52), in which we semi-quantitatively determined the average optical density values.

### Statistical Analysis

Differences between groups were assessed by ANOVA. In all cases, *P* < 0.05 was considered statistically significant. We drew a receiver operator characteristic (ROC) curve to calculate the area under curve (AUC) to discriminate CRSwNP patients from normal subjects. SPSS 16.0 for Windows (IBM, Chicago, USA) was used for ROC analyses and other statistical analyses were performed using GraphPad Prism 7.0 software (GraphPad Software, La Jolla, CA).

## Results

### Integrative Analysis of DEGs in CRSwNP Samples From 4 GEO Datasets

**Figure 2:**
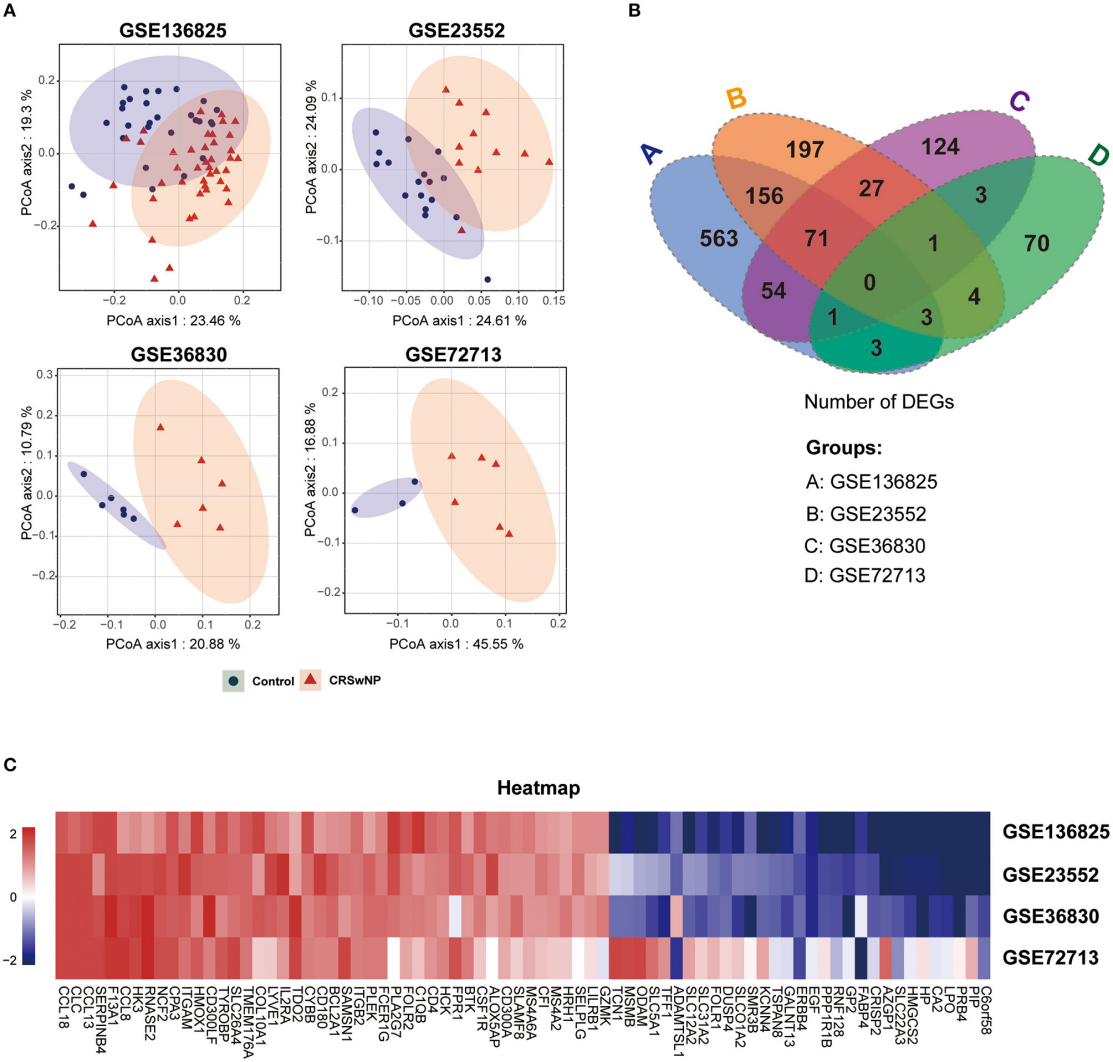
The identification of co-DEGs in four GEO datasets of CRSwNP. **(A)** Principal coordinate analysis (PCoA) of gene expression in CRSwNP and healthy control subjects in four GEO datasets. **(B)** Venn diagram showing the number of integrated genes among the four GEO datasets. **(C)** Gene expression heatmap of the 76 co-DEGs in CRSwNP compared to healthy controls.

To avoid a high proportion of false positives in an individual dataset, multiple-dataset integration was necessary for obtaining reliable results to further investigate the complex molecular mechanisms of CRSwNP. We performed background correction and standardization to reduce variability in four GEO datasets and PCoA, a dimension reduction technique, to present visual coordinates of similarity or differences between the CRSwNP and healthy controls from GEO data (Zhang et al., 2019). PCoA of gene expression in each of the four datasets (GSE136825, GSE36830, GSE23552, and GSE72713) revealed that the samples clustered into two distinct groups (Figure 2A).

We divided the genes into different categories according to their biotype (https://www.ncbi.nlm.nih.gov/gene), and Volcano plots were used to display the gene expression data and *p*-value statistics of each of the datasets (Supplementary Figure 1A). We identified the genes that were significantly differentially expressed (|log_2_FC| > 1, adjusted *p* < 0.05) in nasal polyps compared to control tissues (Supplementary Figure 1B). Then, we integrated the DEGs and identified 76 co-DEGs, including 45 upregulated genes and 31 downregulated genes, derived from the intersections of any three of the four GEO datasets (Figure 2B and Supplementary Table 1). A cluster heatmap was used to visualize the changes in up- and downregulated genes among 76 co-DEGs (Figure 2C), and details of the 76 co-DEGs are shown in Supplementary Table 1.

### GO Functional Enrichment and KEGG Pathway Analyses of co-DEGs in CRSwNP

**Figure 3:**
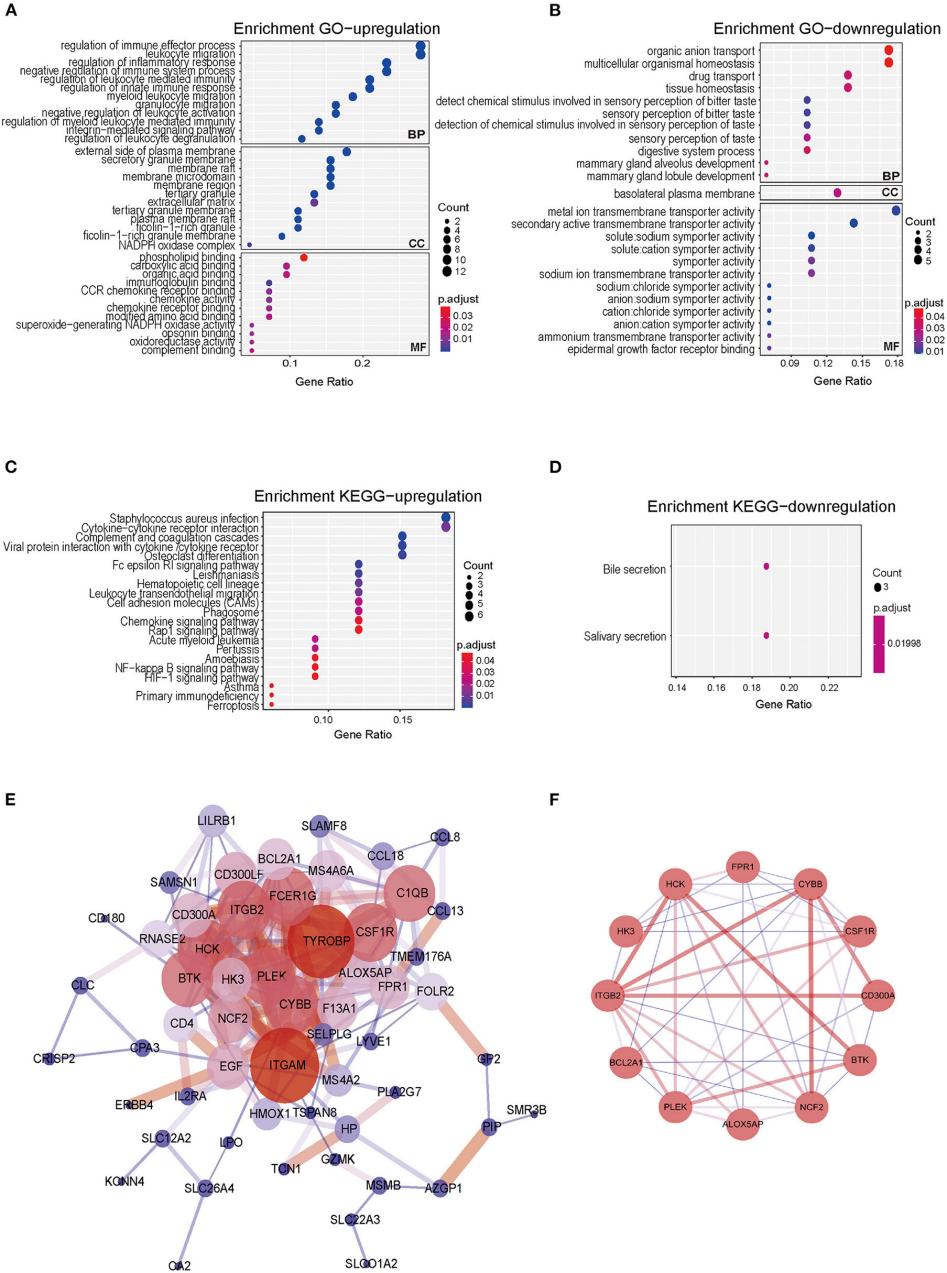
Gene Ontology, KEGG pathway, PPI network of co-DEGs, and hub gene identification analyses in CRSwNP. **(A,B)** Bubble chart showing enriched GO terms for **(A)** upregulated co-DEGs and **(B)** downregulated co-DEGs. **(C,D)** Bubble chart showing enriched KEGG pathways for **(C)** upregulated co-DEGs and **(D)** downregulated co-DEGs. *P*-values < 0.05 and *q*-values< 0.2 were considered statistically significant. **(E)** PPI networks of the 76 co-DEGs from the four GEO datasets of CRSwNP. The node color represents the degree of proteins, and the edge color represents the combined score of proteins. Red represents high, and blue represents low. **(F)** The hub genes of PPI networks.

To explore the potential functions of co-DEGs, we performed GO functional enrichment and KEGG pathway analyses (*p* < 0.05 and *q* < 0.2). Notably, the BP terms associated with the upregulated genes were regulation of immune effector process, leukocyte migration, regulation of inflammatory response, negative regulation of immune system process, and regulation of leukocyte-mediated immunity. The CC terms associated with the upregulated genes were the external side of plasma membrane, secretory granule membrane, and membrane raft. The MF terms associated with the upregulated genes were phospholipid binding, carboxylic acid binding, organic acid binding, G protein-coupled receptor binding, and cytokine receptor binding (Figure 3A). In addition, downregulated genes were also strongly associated with the BP terms organic anion transport, multicellular organismal homeostasis, drug transport, and tissue homeostasis. For downregulated genes, basolateral plasma membrane was found to be the dominant CC term. The significantly enriched MF terms associated with the downregulated genes were metal ion transmembrane transporter activity, secondary active transmembrane transporter activity, and active transmembrane transporter activity (Figure 3B).

In the KEGG pathway analysis, the upregulated genes were mainly related to *Staphylococcus aureus* infection, cytokine-cytokine receptor interactions, complement and coagulation cascades, and viral protein interactions with cytokines and cytokine receptors (Figure 3C), and the downregulated genes were mainly involved in bile secretion and salivary secretion (Figure 3D). The results of GO and KEGG pathway enrichment analyses are also shown in Supplementary Tables 2–4.

### PPI Networks of co-DEGs and Hub Genes in CRSwNP

To further investigate the biological functions of the co-DEGs, we constructed PPI networks according to the 76 co-DEGs in CRSwNP (Figure 3E). The PPI networks contained 57 nodes and 202 edges, and the isolated genes without interactions were removed. The MCODE algorithm was further applied to identify hub genes that were densely associated with each other in the network (Figure 3F). We found that 12 hub genes including Arachidonate 5-Lipoxygenase Activating Protein (*ALOX5AP*), Bcl-2-related protein A1 (*BAL2A1*), Tyrosine-protein kinase BTK (*BTK*), Cytochrome b-245 heavy chain (*CYBB*), Neutrophil cytosol factor 2 (*NCF2*), Tyrosine-protein kinase HCK (*HCK*), Hexokinase-3 (*HK3*), Macrophage colony-stimulating factor 1 receptor (*CSF1R*), Pleckstrin (*PLEK*), CMRF35-like molecule 8 (*CD300A*), Integrin beta-2 (*ITGB2*), and fMet-Leu-Phe receptor (*FPR1*) might play prominent roles in interacting with each other in the PPI network, which indicated that these 12 genes might be core molecules in the development of CRSwNP (Supplementary Tables 5, 6). The 12 genes screened from the PPI network were also related to neutrophil-mediated immunity, positive regulation of the innate immune response, positive regulation of the defense response, and *Staphylococcus aureus* infection as determined by GO functional enrichment and KEGG pathway analyses.

### Validation of Hub Genes

**Figure 4:**
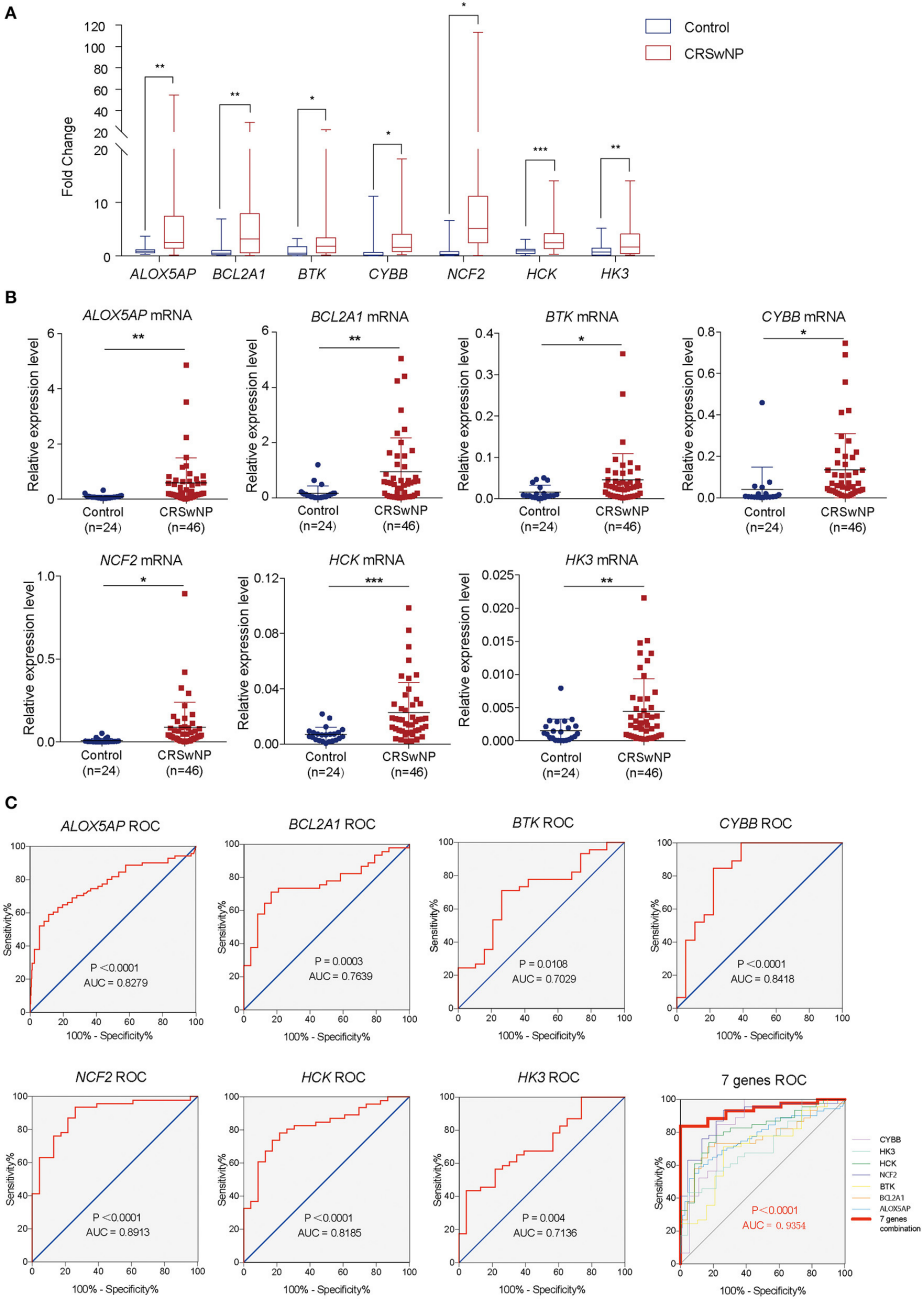
The gene expression levels and diagnostic values of 7 hub genes in CRSwNP. **(A)** Fold changes in the *ALOX5AP, BCL2A1, BTK, CYBB, NCF2, HCK*, and *HK3* genes in CRSwNP as determined by RT-qPCR. **(B)** The relative expression levels of the *ALOX5AP, BCL2A1, BTK, CYBB, NCF2, HCK*, and *HK3* genes in CRSwNP. *GAPDH* was used as a reference. **(C)** ROC curves for testing the hub genes *ALOX5AP, BCL2A1, BTK, CYBB, NCF2, HCK, HK3*, and the combination of 7 hub genes as determined by RT-qPCR. *P < 0.05, **P < 0.01, ***P < 0.001.

To further validate the results of bioinformatics analysis, the gene expression levels of the 12 hub genes from PPI network (*ALOX5AP, BCL2A1, BTK, CYBB, NCF2, HCK, HK3, CSF1R, PLEK, CD300A, ITGB2*, and *FPR1*) in nasal polyps from CRSwNPs and nasal mucosa from healthy controls were determined by RT-qPCR. As illustrated in Figure 4 and Supplementary Figure 2, the expression levels of *ALOX5AP, BCL2A1, BTK, CYBB, NCF2, HCK*, and *HK3* were significantly altered in CRSwNP, as identified by the bioinformatics analysis. The other five genes did not show significantly different expression levels in CRSwNP and healthy control samples. Regarding diagnostic prediction quality, the hub genes *ALOX5AP, BCL2A1, BTK, CYBB, NCF2, HCK*, and *HK3* performed well-according to receiver operator characteristic (ROC) analysis (Figure 4C and Supplementary Table 9). The area under the ROC curves (AUC) of the genes *ALOX5AP, BCL2A1, BTK, CYBB, NCF2, HCK*, and *HK3* are 0.7698, 0.7639, 0.7029, 0.8418, 0.8913, 0.8185, 0.7136, respectively. The AUC of combined detection of the 7 indexes was 0.9354, which was higher than that of each single detection. Both the qPCR and ROC analyses suggest that these seven hub genes could be diagnostic biomarkers for CRSwNP.

**Figure 5:**
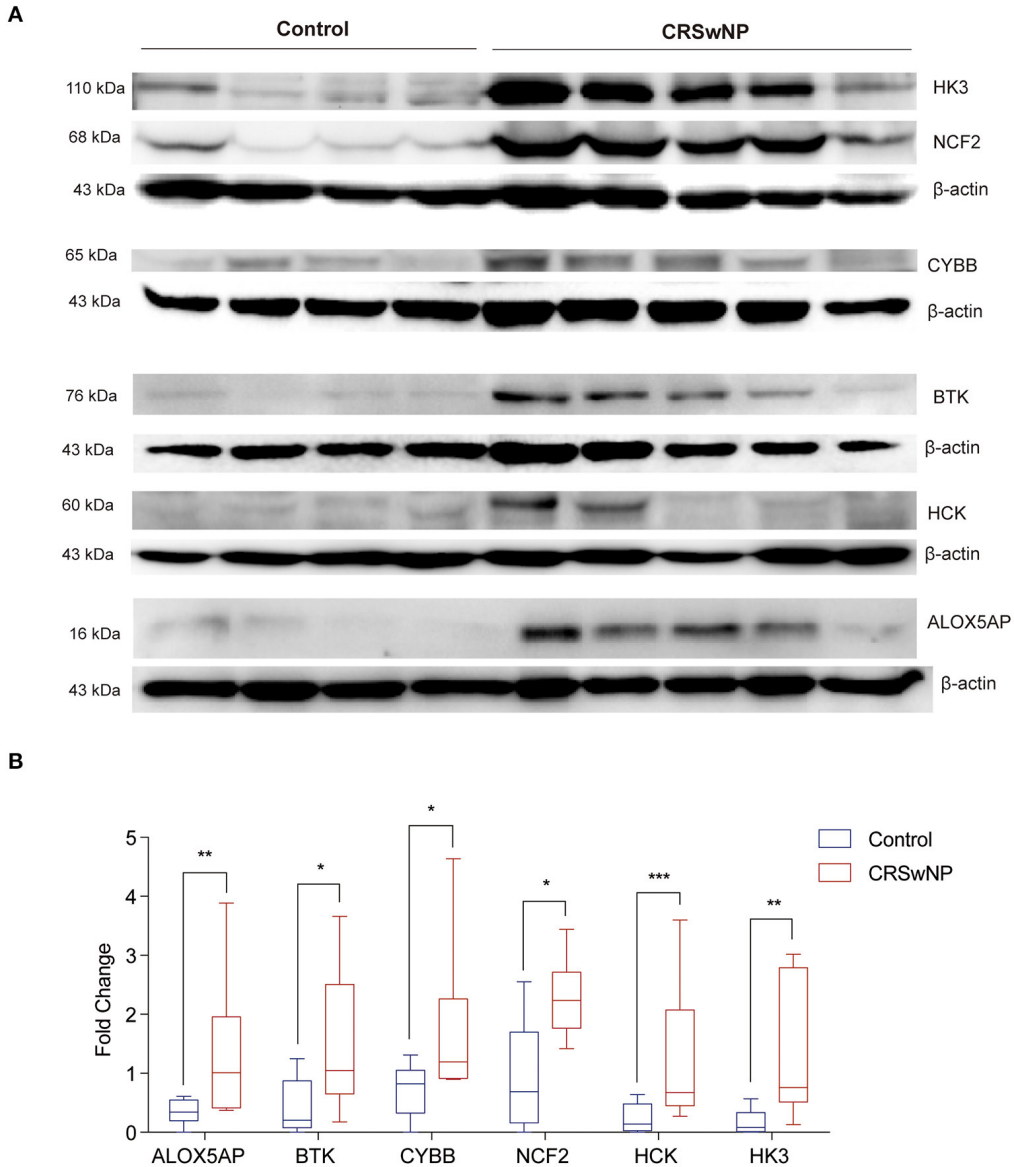
The protein expression levels 6 hub genes were significantly increased in CRSwNP by western blot analysis. **(A)** The expression level of ALOX5AP, BCL2A1, BTK, CYBB, NCF2, HCK, and HK3 proteins in nasal polyps from CRSwNP patients (*n* = 10) and nasal mucosal tissues from healthy control (*n* = 8). **(B)** Fold changes of relative expression ratio of ALOX5AP, BCL2A1, BTK, CYBB, NCF2, HCK, and HK3 compare to β-actin in CRSwNP and healthy controls. The expression level of β-actin was used as a reference. *P < 0.05, **P < 0.01, ***P < 0.001.

**Figure 6:**
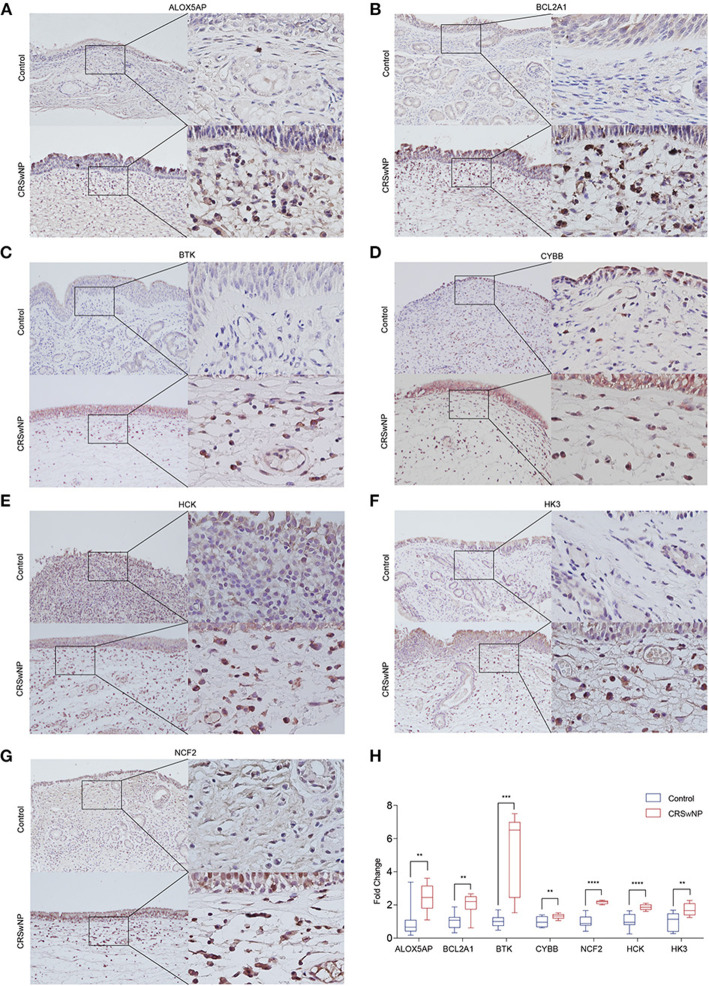
The protein expression levels 7 hub genes were significantly increased in CRSwNP by immunohistochemistry staining. **(A–G)** The expression level and location distribution of ALOX5AP, BCL2A1, BTK, CYBB, NCF2, HCK, and HK3 proteins in nasal polyps from CRSwNP patients (*n* = 11) and nasal mucosal tissues from healthy control (*n* = 7). **(H)**, Fold changes of average optical density value in the ALOX5AP, BCL2A1, BTK, CYBB, NCF2, HCK, and HK3 protein expression in CRSwNP and healthy controls. **P < 0.01, ***P < 0.001, ****P < 0.0001.

Next, we identified the protein level of the seven hub genes (ALOX5AP, BCL2A1, BTK, CYBB, NCF2, HCK, and HK3) from nasal polyps from CRSwNPs and nasal mucosa from healthy controls. The western blot results showed the expression level of ALOX5AP, BTK, CYBB, NCF2, HCK, and HK3 in CRSwNP was significantly increased in nasal polyps compared to control subjects (Figure 5). Moreover, the immunohistochemistry stain results also demonstrated that the protein level of ALOX5AP, BCL2A1, BTK, CYBB, NCF2, HCK, and HK3 were significantly increased in nasal polyps compared to control subjects (Figure 6). We found that ALOX5AP, BCL2A1, BTK, CYBB, NCF2, HCK, and HK3 were broadly expressed on both epithelial layer and stromal layer in nasal polyp tissues.

### Prediction of Potential Novel Drugs for the Treatment of CRSwNP by CMap

To identify potential drugs for CRSwNP treatment, we introduced 45 upregulated co-DEGs and 31 downregulated co-DEGs from the four GEO datasets into the CMap database and matched them with targeted drug therapies. The top 15 most significant small molecules (Alfadolone, Hydralazine, SC-560, Iopamidol, Iloprost, Clorgiline, Cefotetan, Etynodiol, Disulfiram, Ketotifen, Florfenicol, Clidinium bromide, Ramifenazone, Nafcillin, Bepridil) and their enrichment value are listed in Table 2. These drug repurposing candidates could target co-DEGs in CRSwNP and then affect the expression and function of genes. This provides a novel perspective to explore potential precise targeted drugs for CRSwNP treatment. Further experiments are needed to confirm the efficacy of these drug candidates in CRSwNP.

**Table 2:** Results of CMap analysis of co-DEGs in CRSwNP.

**Rank**	**CMap name**	**Mean**	***N***	**Enrichment**	***P*-value**	**CID**	**Molecular formula**	**Group**
1	Alfadolone	0.715	3	0.942	0.00024	9798416	C21H32O4	Approved
2	Hydralazine	0.27	6	0.752	0.00058	3637	C8H8N4	Approved
3	SC-560	0.631	3	0.905	0.00176	4306515	C17H12ClF3N2O	Experimental
4	Iopamidol	−0.255	4	−0.825	0.00177	65492	C17H22I3N3O8	Approved
5	Iloprost	−0.686	3	−0.899	0.00194	5311181	C22H32O4	Approved
6	Clorgiline	0.287	4	0.8	0.003	4380	C13H15Cl2NO	Experimental
7	Cefotetan	0.484	3	0.877	0.00355	53025	C17H17N7O8S4	Approved
8	Etynodiol	−0.319	4	−0.793	0.00374	14687	C20H28O2	Experimental
9	Disulfiram	0.576	5	0.722	0.00378	3117	C10H20N2S4	Approved
10	Ketotifen	−0.322	4	−0.782	0.00462	3827	C19H19NOS	Approved
11	Florfenicol	0.242	4	0.764	0.00585	114811	C12H14Cl2FNO4S	Approved
12	Clidinium bromide	−0.303	4	−0.753	0.00746	19004	C22H26BrNO3	Approved
13	Ramifenazone	0.62	4	0.731	0.01038	5037	C14H19N3O	Experimental
14	Nafcillin	0.454	4	0.725	0.0115	8982	C21H22N2O5S	Approved
15	Bepridil	0.284	4	0.724	0.01162	2351	C24H34N2O	Approved

*CMap, Connectivity Map; CID, Compound ID*.

## Discussion

Previous studies were limited to individual datasets or incorrect combinations, while our study integrated all the available public GEO databases of CRSwNP. The CRSwNP groups were independent of the normal control groups in each of the four datasets as determined by PCoA. We identified 76 co-DEGs (45 upregulated and 31 downregulated) among all the GEO data. The PPI network provided an overview illustration of the associations among the 76 co-DEGs, and we identified 7 hub genes not only by mRNA level, also by protein expression level that might be biomarkers and key regulators of CRSwNP pathogenesis.

ALOX5AP is an essential regulator of the biosynthesis of leukotriene B4 (Haeggstrom, 2018). Previous studies on the genome-wide gene expression profile of CRSwNP showed increased *ALOX5AP* gene expression levels in the nasal polyps of patients with aspirin-intolerant asthma (Sekigawa et al., 2009) and decreased methylation levels of ALOX5AP in a genome-wide methylation profile of nasal polyps (Cheong et al., 2011). BTK, a member of the Tec family of tyrosine kinases, has been indicated to play crucial roles in B cell development and signal transduction downstream of the high-affinity receptor for IgE (FcεR) on mast cells and basophils in an ovalbumin-induced mouse model of asthma (Phillips et al., 2016). However, there have been no studies on BTK in chronic nasal diseases. NCF2 (also called p67phox) is a subunit of the multiprotein NADPH oxidase complex, which is an essential component of the innate immune response responsible for effective superoxide production in neutrophils (Thomas, 2017). Another study from our group previously found that p67phox expression was significantly increased in nasal polyp tissue compared with control mucosal tissue (Zheng et al., 2020). A study using a nitric oxide polymerase chain reaction array showed significant upregulation of NCF2 expression in CRS patients who were both *Staphylococcus aureus* biofilm-positive and polyp-positive compared to control subjects (Jardeleza et al., 2013). HCK, a member of the Src family of tyrosine kinases, acts as a key regulator of gene expression in alternatively activated monocytes/macrophages (Bhattacharjee et al., 2011). Similar to NCF2, CYBB (often referred to as p91phox or NOX2) has also been found to be upregulated in CRSwNP (Zheng et al., 2020). HK3 played a functional role in acute promyelocytic leukemia, non-small lung cancer, and colorectal cancer (Federzoni et al., 2014; Wolf et al., 2016; Pudova et al., 2018; Tuo et al., 2020). BCL2A1 is a member of the BCL-2 family of antiapoptotic proteins that is induced by mucin 1 transmembrane C-terminal (MUC1-CT) via the NF-κB p65-dependent signaling pathway (Hiraki et al., 2018). MUC1 has been identified as an anti-inflammatory molecule that could inhibit bacteria- and virus-induced inflammation in airways (Kim and Lillehoj, 2008; Li et al., 2010; Kyo et al., 2012). MUC1-CT also participates in the corticosteroid response in the treatment of CRSwNP (Milara et al., 2015), but relationships between BCL2A1 and CRSwNP remain unknown. Our study has proved the increased expression of ALOX5AP, BCL2A1, BTK, CYBB, NCF2, HCK, and HK3 by mRNA and protein levels in nasal polyps.

Currently, colonization by fungi and bacteria, alterations in mucociliary clearance, abnormalities in the sinonasal epithelial cell barrier and tissue remodeling combined with host innate and adaptive immune responses are known to contribute to the chronic inflammatory and tissue-deforming processes characteristic of CRS (Stevens et al., 2015a). In our study, GO and KEGG results showed that upregulated genes were predominantly enriched for the immune effector process, leukocyte migration, regulation of the inflammatory response, negative regulation of the immune system process, and regulation of leukocyte-mediated immunity. Dysregulation of these processes indicated that increasing exposure to pathogenic and colonized bacteria ultimately caused complicated downstream immune responses and chronic inflammation during the formation and development of nasal polyps. Additionally, downregulated genes were enriched for multicellular organismal homeostasis and tissue homeostasis, which reflected the destruction of the sinonasal epithelial cell barrier and tissue remodeling in CRSwNP. KEGG pathway analysis demonstrated that the upregulated genes were mainly related to *Staphylococcus aureus* infection and cytokine-cytokine receptor interactions. The above pathways are critical biological processes for pathogen invasion, immune effector and inflammatory responses, and tissue homeostasis disorder. It is worth noting that dysregulation of these processes eventually leads to a severe immune response and the formation of nasal polyps.

Although previous studies involved bioinformatic analysis of mRNAs and lncRNAs in CRSwNP (Liu et al., 2019; Zhou et al., 2020), one study included only 12 CRSwNP patients and 9 healthy controls. The other study used nasal tissue data combined with primary human basal cells cultured in an air-liquid interface system, which might be different from nasal polyp tissue. Our study included 65 CRSwNP patients and 54 healthy controls, representing the largest CRSwNP study to date. Additionally, we identified new genes that might be involved in the pathogenesis of CRSwNP. Moreover, we used the CMap database to identify drug repurposing candidates potentially targeting the co-DEGs derived from the four GEO datasets. Repurposing drugs with higher enrichment scores are more likely to reverse the gene expression changes seen in CRSwNP than those with lower enrichment scores. This work may help to develop new drugs for CRSwNP treatment. Ketotifen is a cycloheptathiophene blocker of histamine H1 receptors and inflammatory mediator release that has been widely used in the treatment of allergic rhinitis, asthma and allergic conjunctivitis, and its common side effects include tiredness, dry mouth, and nausea. Clidinium bromide is a synthetic anticholinergic agent associated with antispasmodic and antisecretory effects on the gastrointestinal tract and has the side effects of dry mouth, dry skin and flushed face. Cefotetan and nafcillin are broad-spectrum cephalosporin antibiotics might rarely cause allergic reactions that include a rash, systemic papules, urticaria, pruritus, and fever. These drugs have not yet been reported as therapies for CRSwNP. Traditionally, CRSwNP treatments include nasal saline irrigation, intranasal corticosteroids, oral antibiotics, oral corticosteroids, and surgery depending on both the site and symptoms of disease (Kariyawasam and Scadding, 2011; Lee, 2015). With the advancement of the concept of CRSwNP endotypes, the management of precision medicine and reductions in recurrence are evolving (Kim and Cho, 2017). Endotyping helps physicians to determine optimal primary therapeutic modality and predict treatment outcomes and risks for comorbidities. Biologics in CRSwNP mainly focus on targeting the type 2 cytokines such as IL-4, IL-5, IL-13, as well as IgE. Combining our study with previous studies, the hub genes increased in CRSwNP might be served as biomarkers. Our study provides new insights which will shift drug discovery toward the personalized and precision medicine treatment approach to enhance CRSwNP therapies. Therefore, further research is needed to explore the potential of new targeted drugs in CRSwNP treatment.

## Conclusion

In summary, by comprehensively analyzing gene expression profiles, sequencing data from four CRSwNP GEO datasets, identifying key genes and important pathways, and predicting the repurposing drugs for CRSwNP treatment, our study elucidated molecular mechanisms underlying the occurrence and development of CRSwNP to explain its pathogenesis and aid in diagnosis from the perspective of bioinformatics. Our study successfully identified 7 potential genes as key regulators and predicted a series of repurposing drugs to expand CRSwNP treatment. However, more experimental validation is necessary before these data can be translated into the clinic.

## Data Availability Statement

The original contributions presented in the study are included in the article/Supplementary Material, further inquiries can be directed to the corresponding author/s.

## Ethics Statement

The studies involving human participants were reviewed and approved by this study was approved by the Ethics Committee of Beijing TongRen Hospital, Capital Medical University (TRECKY2019-050). All subjects who participated in this research provided written informed consent. The patients/participants provided their written informed consent to participate in this study.

## Author Contributions

LZ and XW conceived and designed the project. YH performed the integrated analysis under the guidance of YZ, ZY, and KD. PW helped collect the GEO database data. YH and YZ did the experiments with the help of PW and YL. YH and YZ wrote the manuscript. All authors contributed to the article and approved the submitted version.

## Conflict of Interest

The authors declare that the research was conducted in the absence of any commercial or financial relationships that could be construed as a potential conflict of interest. The reviewer DW declared a shared affiliation, with no collaboration, with the authors YH, YZ, PW, KD, XW and LZ to the handling Editor.
