# Supplementary material for: Transcriptomic Signatures and Functional Network Analysis of Chronic Rhinosinusitis With Nasal Polyps
Source: Front Genet. 2021 Feb 2;12:609754. doi: 10.3389/fgene.2021.609754 (PMC7884819; doi:10.3389/fgene.2021.609754)
Supplement: Supplementary file 1 [file Data_Sheet_1.DOCX]

Supplementary Material

# Supplementary Figures and Tables

## Supplementary Figures


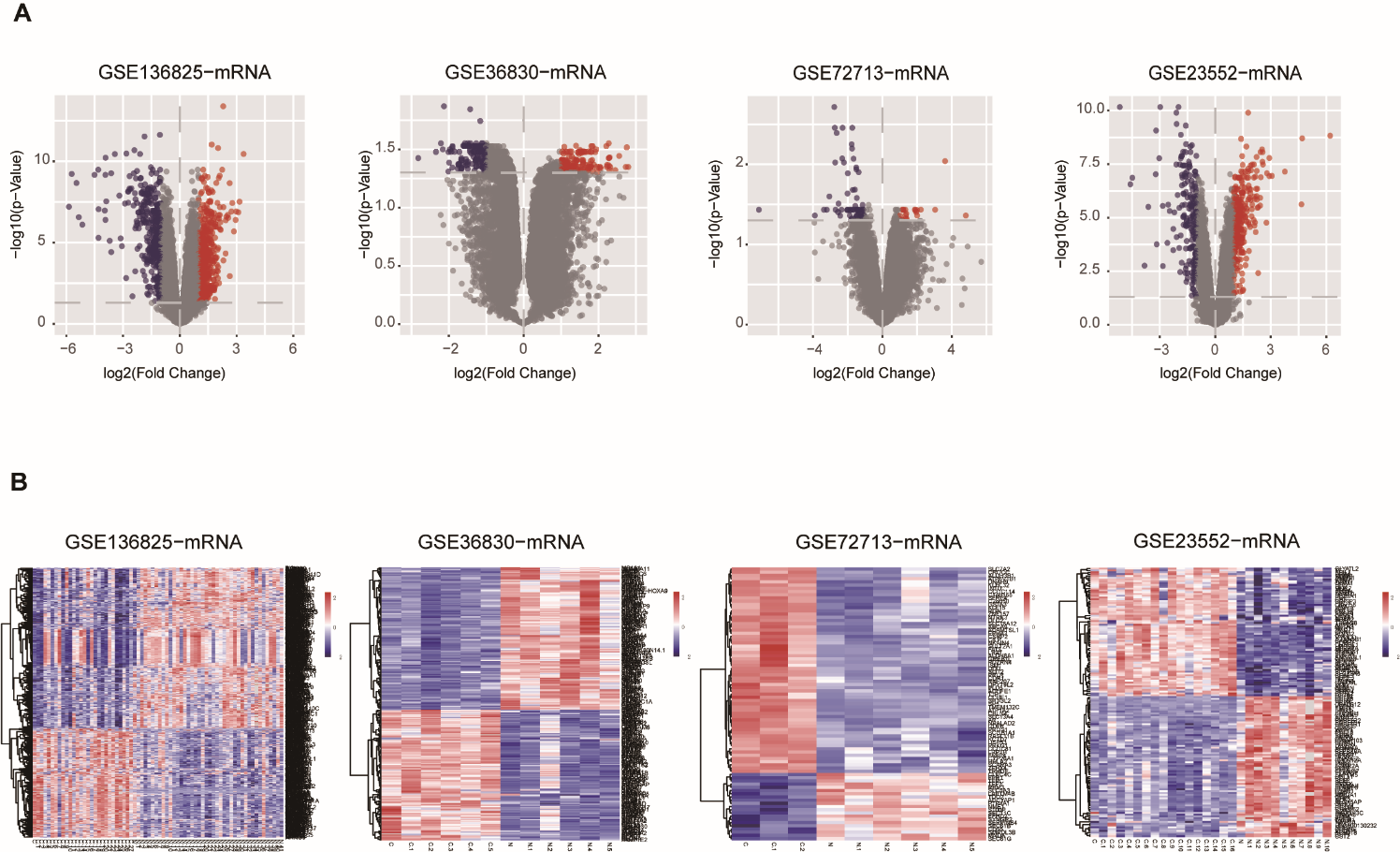


**Supplementary Figure 1.** Identification of differentially expressed mRNAs in CRSwNP. **A**, Volcano plots of differentially expressed mRNAs in the 4 GEO datasets. **B**, Heatmaps of differentially expressed mRNAs in the 4 GEO datasets. Genes with |log_2_FC| > 1 and adjusted p-values < 0.05 are shown in volcano plots. Red represents upregulation in CRSwNP, blue represents downregulation in CRSwNP, and gray represents no significant change.


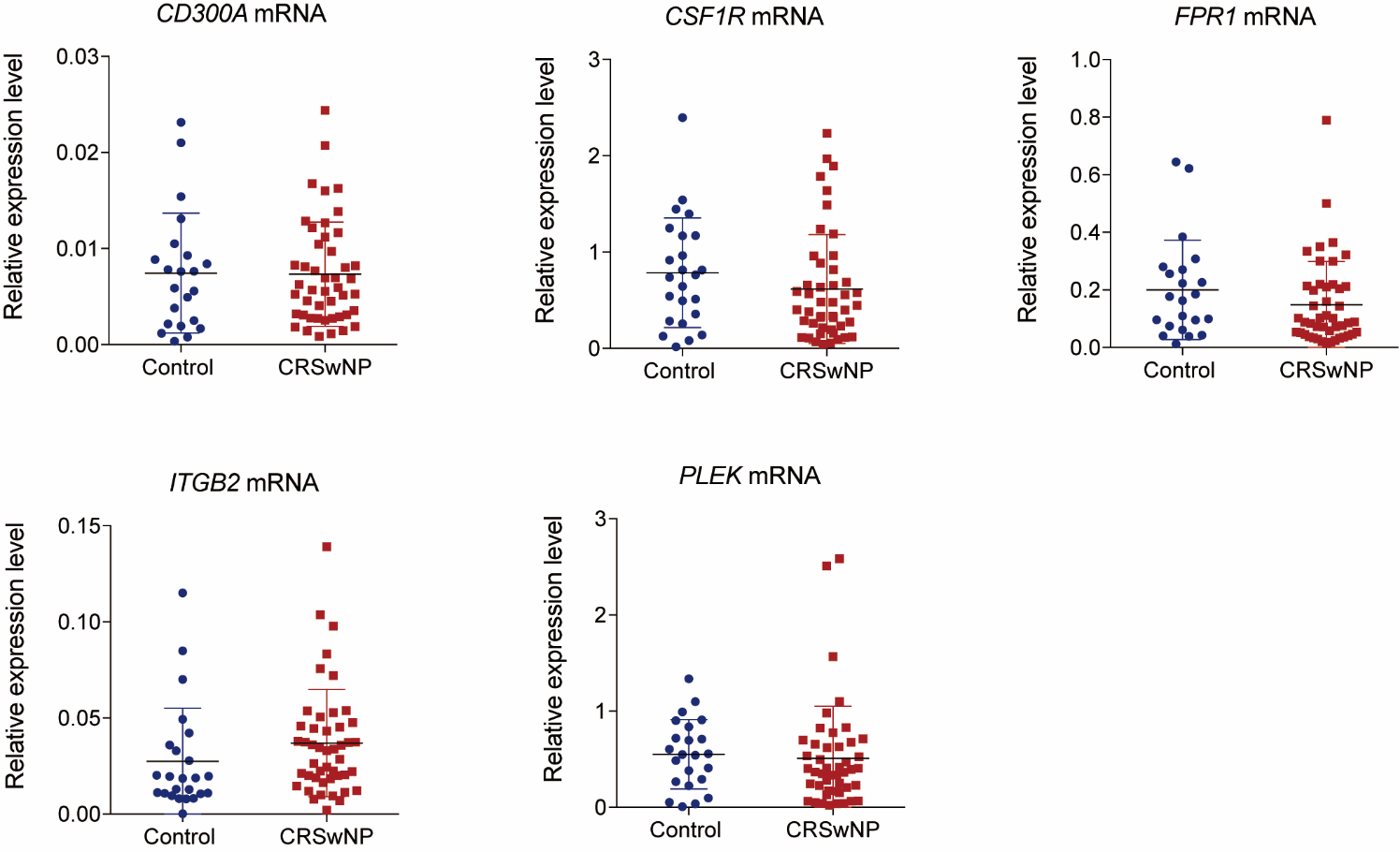


**Supplementary Figure 2.** The expression levels of 5 hub genes in CRSwNP. The expression levels of the *CD300A, CSF1R, FPR1, ITGB2* and *PLEK* genes in the control (n=24) and CRSwNP (n=46) groups as determined by RT-qPCR; *GAPDH* served as the reference.

## Supplementary Tables

**Table S1.** Seventy-six co-DEGs in CRSwNP from 4 GEO datasets

|  | **GSE136825** | | | | **GSE36830** | | | | **GSE23552** | | | **GSE72713** | | |
| --- | --- | --- | --- | --- | --- | --- | --- | --- | --- | --- | --- | --- | --- | --- |
| **Gene name** | **Log_2_FC** | | **adj. p-value** |  | **Log_2_FC** | | **adj. p-value** |  | **Log_2_FC** | **adj. p-value** |  | **Log_2_FC** | **adj. p-value** |  |
| FPR1 | 1.819321077 | | 0.0000165 | up | -0.092808462 | | 0.931505118 | down | 1.787836726 | 7.02672E-08 | up | 1.871935803 | 0.036872596 | up |
| ADAMTSL1 | -1.00142504 | | 0.0000604 | down | 0.926315802 | | 0.229937919 | up | -1.156083076 | 1.04058E-05 | down | -1.560819216 | 0.033009761 | down |
| FABP4 | -1.487174917 | | 0.0000433 | down | -0.204821282 | | 0.701015889 | down | -1.308703506 | 0.001515918 | down | -3.203725924 | 0.036872596 | down |
| TDO2 | 0.941943449 | | 0.00025894 | up | 2.219102304 | | 0.044739252 | up | 1.053250433 | 0.000103828 | up | 2.006599719 | 0.043524397 | up |
| SERPINB4 | 2.981391666 | | 0.000000122 | up | 1.914260117 | | 0.033316019 | up | 1.26392599 | 0.070667828 | up | 4.812237127 | 0.043524397 | up |
| SLC22A3 | -2.723678161 | | 7.26E-09 | down | -1.255198728 | | 0.03755121 | down | -1.859165202 | 1.34858E-09 | down | -0.760239021 | 0.203936032 | down |
| CCL18 | 1.825732538 | | 0.008755352 | up | 4.491923109 | | 0.014281489 | up | 6.206296494 | 1.4859E-09 | up | 4.747305461 | 0.353618897 | up |
| CLC | 1.619426951 | | 0.00551061 | up | 3.997576045 | | 0.033102324 | up | 4.694315807 | 1.97356E-09 | up | 5.701352644 | 0.164951716 | up |
| NCF2 | 1.334078764 | | 0.0000205 | up | 1.809691266 | | 0.029588741 | up | 2.55918974 | 8.65246E-09 | up | 1.895289119 | 0.212913878 | up |
| ALOX5AP | 1.440918895 | | 0.0000664 | up | 1.034183564 | | 0.044739252 | up | 2.43685068 | 8.65246E-09 | up | 0.169101988 | 0.834008815 | up |
| F13A1 | 2.431012465 | | 0.000000422 | up | 2.274837736 | | 0.02801553 | up | 2.920825781 | 1.63156E-08 | up | 2.153592199 | 0.0823035 | up |
| RNF128 | -2.263933711 | | 3.64E-08 | down | -1.299718058 | | 0.033102324 | down | -1.370584874 | 4.20636E-08 | down | -0.444220611 | 0.282853417 | down |
| CCL13 | 1.788574301 | | 0.00197638 | up | 4.116178254 | | 0.013468242 | up | 3.758362585 | 7.02672E-08 | up | 4.620200242 | 0.266575839 | up |
| LPO | -4.308753653 | | 3.28E-10 | down | -1.561733645 | | 0.029588741 | down | -3.217723751 | 9.34344E-08 | down | - | - | - |
| ITGAM | 1.401706573 | | 0.0000766 | up | 1.792712618 | | 0.033102324 | up | 2.165381663 | 1.20344E-07 | up | 1.536325768 | 0.345975047 | up |
| CD180 | 1.105166772 | | 0.001955115 | up | 1.535344343 | | 0.033102324 | up | 2.088707523 | 1.33526E-07 | up | 1.357314888 | 0.156973426 | up |
| PIP | -5.70938593 | | 5.95E-10 | down | -1.082627742 | | 0.041055631 | down | -4.480205637 | 1.3635E-07 | down | 0.932789965 | 0.676636214 | up |
| C6orf58 | -6.361924222 | | 9.26E-10 | down | -1.372113345 | | 0.042205653 | down | -4.588200233 | 2.75305E-07 | down | -0.989888012 | 0.408705249 | down |
| HCK | 1.528099745 | | 0.00000534 | up | 1.184785617 | | 0.044638632 | up | 1.448492368 | 3.58292E-07 | up | 1.353704906 | 0.207863118 | up |
| COL10A1 | 2.776469624 | | 3.64E-08 | up | 1.831268568 | | 0.039034412 | up | 1.127223636 | 4.41392E-07 | up | 0.622703731 | 0.426685395 | up |
| IL2RA | 1.319351659 | | 0.0000608 | up | 1.587301491 | | 0.042487344 | up | 2.208539049 | 4.47936E-07 | up | 1.113548118 | 0.257969815 | up |
| CFI | 1.183562897 | | 0.00000312 | up | 1.155928524 | | 0.04144661 | up | 1.481513151 | 6.93146E-07 | up | 0.734022778 | 0.263712231 | up |
| CRISP2 | -3.432566753 | | 3.56E-11 | down | -1.22217813 | | 0.029588741 | down | -1.339492837 | 1.11662E-06 | down | -0.145692898 | 0.872691596 | down |
| LYVE1 | 1.446722986 | | 0.002140647 | up | 1.741468779 | | 0.044739252 | up | 2.428752812 | 1.17516E-06 | up | 0.650987109 | 0.644343864 | up |
| BCL2A1 | 1.194269713 | | 0.003110745 | up | 1.668083605 | | 0.042858566 | up | 1.840703994 | 1.17516E-06 | up | 1.334081468 | 0.461454598 | up |
| PLA2G7 | 2.118825608 | | 6.63E-08 | up | 1.518698773 | | 0.033102324 | up | 1.907031532 | 1.49026E-06 | up | 0.178983701 | 0.896282358 | up |
| DUSP4 | -1.486563327 | | 0.000000598 | down | -1.040015352 | | 0.040352104 | down | -1.135314027 | 1.76243E-06 | down | 0.577007252 | 0.393676905 | up |
| BTK | 1.132615892 | | 0.000338514 | up | 1.123269677 | | 0.043259689 | up | 1.628051896 | 2.27582E-06 | up | 1.418941358 | 0.138682429 | up |
| EGF | -1.676521681 | | 0.00000123 | down | -1.61504685 | | 0.029359979 | down | -1.630492029 | 2.45395E-06 | down | -0.227915828 | 0.69838879 | down |
| AZGP1 | -3.963541117 | | 0.000000107 | down | -1.139827912 | | 0.033316019 | down | -2.840568087 | 2.80416E-06 | down | 1.627656759 | 0.414357484 | up |
| ERBB4 | -1.066617499 | | 0.000000506 | down | -1.043779316 | | 0.039705169 | down | -1.272188732 | 2.80416E-06 | down | -1.372633754 | 0.086770608 | down |
| C1QB | 2.146876751 | | 0.00000056 | up | 1.41333763 | | 0.046883878 | up | 1.231104089 | 4.02173E-06 | up | 0.73551648 | 0.491385566 | up |
| SELPLG | 1.261713706 | | 0.0000297 | up | 1.121170642 | | 0.039034412 | up | 1.464213827 | 4.09364E-06 | up | 0.173051182 | 0.682306393 | up |
| SAMSN1 | 1.260712923 | | 0.00000284 | up | 1.16987221 | | 0.033316019 | up | 1.503044373 | 4.2714E-06 | up | 1.991403946 | 0.092769402 | up |
| PLEK | 1.048983615 | | 0.002622985 | up | 1.605869506 | | 0.033102324 | up | 1.703010585 | 5.14686E-06 | up | 1.445171344 | 0.163408157 | up |
| FOLR1 | -1.818676117 | | 0.0000657 | down | -1.098940879 | | 0.045259029 | down | -1.002110182 | 5.54091E-06 | down | 0.879890768 | 0.436023739 | up |
| MS4A2 | 1.098373895 | | 0.000103499 | up | 1.27778873 | | 0.033122061 | up | 1.32298394 | 5.54387E-06 | up | 0.649266224 | 0.421647084 | up |
| FOLR2 | 1.799854873 | | 0.0000462 | up | 1.383939798 | | 0.044638632 | up | 1.419990836 | 6.87114E-06 | up | 1.017423515 | 0.23732399 | up |
| HK3 | 1.273907927 | | 0.002586481 | up | 2.059817028 | | 0.045799902 | up | 1.942360461 | 8.36131E-06 | up | 2.493356428 | 0.189404686 | up |
| ITGB2 | 1.573941499 | | 0.000000756 | up | 1.403850871 | | 0.039034412 | up | 1.457558668 | 8.8717E-06 | up | 1.394883634 | 0.168176896 | up |
| CA2 | -3.932128667 | | 6.79E-10 | down | -1.489294604 | | 0.02801553 | down | -1.817493848 | 8.91294E-06 | down | -0.031058273 | 0.975308797 | down |
| SLC12A2 | -1.959195753 | | 7.62E-09 | down | -1.158478344 | | 0.018028513 | down | -0.698604755 | 9.92573E-06 | down | 1.021859833 | 0.178197223 | up |
| CD300 LF | 1.341806727 | | 0.0000379 | up | 2.298819071 | | 0.036976524 | up | 1.678723119 | 1.19473E-05 | up | 1.39554356 | 0.285396181 | up |
| CPA3 | 1.854675754 | | 0.00000103 | up | 1.741357156 | | 0.033102324 | up | 1.828651809 | 1.27711E-05 | up | 1.889922516 | 0.146389756 | up |
| SLAMF8 | 1.340214518 | | 0.00000175 | up | 1.587224252 | | 0.033122061 | up | 1.174356252 | 1.23904E-05 | up | 0.595629987 | 0.421426026 | up |
| FCER1G | 1.325269724 | | 0.0000748 | up | 1.586656877 | | 0.041335865 | up | 1.448086739 | 1.45516E-05 | up | 1.432333617 | 0.103221114 | up |
| CCL8 | 1.137119446 | | 0.0192105 | up | 2.553072989 | | 0.035970379 | up | 1.949790074 | 1.66835E-05 | up | 2.159177788 | 0.179022483 | up |
| RNASE2 | 1.099961828 | | 0.000863841 | up | 2.294899181 | | 0.04752558 | up | 1.905516958 | 2.02789E-05 | up | 2.321689866 | 0.151739443 | up |
| SLC5A1 | -1.874673237 | | 0.000103447 | down | -1.396089244 | | 0.034271657 | down | -0.565014897 | 2.14551E-05 | down | 1.575717791 | 0.102273592 | up |
| PPP1R1B | -3.194263832 | | 1.29E-08 | down | -1.239772903 | | 0.030050196 | down | -1.330316912 | 2.8486E-05 | down | 0.443085998 | 0.781646359 | up |
| GALNT13 | -1.725962294 | | 0.000000119 | down | -1.435224574 | | 0.033102324 | down | -1.093006 | 3.15429E-05 | down | -0.117831133 | 0.909913933 | down |
| HRH1 | 1.004620531 | | 0.000859408 | up | 1.463282373 | | 0.033316019 | up | 1.042714156 | 3.3161E-05 | up | 0.660239564 | 0.430076935 | up |
| TYROBP | 1.881316639 | | 0.000000708 | up | 1.304944578 | | 0.044638632 | up | 1.673407175 | 3.5241E-05 | up | 1.833960309 | 0.134145746 | up |
| CD4 | 1.639522966 | | 0.000000555 | up | 1.348391821 | | 0.041335865 | up | 1.38141535 | 3.86893E-05 | up | 1.156331264 | 0.225388079 | up |
| SLC31A2 | -1.442314814 | | 0.000000199 | down | -1.34277517 | | 0.036570614 | down | -0.729540575 | 4.58915E-05 | down | 0.596468258 | 0.425652057 | up |
| CD300A | 1.468684781 | | 0.0000109 | up | 1.242006522 | | 0.041579048 | up | 1.221194502 | 4.54727E-05 | up | 0.952892203 | 0.234414416 | up |
| KCNN4 | -2.722601548 | | 3.51E-08 | down | -1.111518051 | | 0.033122061 | down | -0.943615678 | 6.09436E-05 | down | 1.003272565 | 0.159080765 | up |
| CSF1R | 1.699410549 | | 0.000000237 | up | 1.450586065 | | 0.044608007 | up | 1.287236356 | 6.21006E-05 | up | 0.711712453 | 0.360702736 | up |
| HMOX1 | 2.057125624 | | 1.05E-09 | up | 1.328330702 | | 0.047299066 | up | 1.715831557 | 7.49954E-05 | up | 1.762038344 | 0.106145716 | up |
| HP | -3.885368439 | | 2.91E-08 | down | -1.973188485 | | 0.031590424 | down | -1.805645122 | 8.04435E-05 | down | 0.441621558 | 0.800704504 | up |
| CYBB | 1.905948484 | | 0.00000107 | up | 1.34852912 | | 0.03823491 | up | 1.552710018 | 9.19891E-05 | up | 1.403652451 | 0.102530069 | up |
| LILRB1 | 1.256967298 | | 0.0000713 | up | 1.261714536 | | 0.04948622 | up | 0.993432392 | 0.000108332 | up | 0.461615341 | 0.526633884 | up |
| MSMB | -1.693348161 | | 0.005412438 | down | -1.032023699 | | 0.048931644 | down | -0.261962305 | 0.000149905 | down | 1.939484944 | 0.262873405 | up |
| MS4A6A | 1.428618805 | | 0.00000689 | up | 1.151083938 | | 0.040737848 | up | 1.165956059 | 0.000148574 | up | 0.918165433 | 0.269746469 | up |
| TMEM176A | 1.755583629 | | 0.00000113 | up | 1.662493849 | | 0.033316019 | up | 1.633037048 | 0.00019042 | up | 1.481033666 | 0.09708514 | up |
| TCN1 | -3.123391877 | | 0.00000551 | down | -1.048894856 | | 0.034676582 | down | -0.22033399 | 0.000257744 | down | 3.511284762 | 0.281277192 | up |
| GZMK | 1.242343118 | | 0.000197365 | up | 1.449337322 | | 0.033554906 | up | 0.868296859 | 0.000399574 | up | -0.086789864 | 0.957340403 | down |
| SMR3B | -1.084408747 | | 0.004934514 | down | -1.905438836 | | 0.045264655 | down | -0.907168619 | 0.001052562 | down | 0.268341726 | 0.901332169 | up |
| TFF1 | -1.491383036 | | 0.000616588 | down | -1.672089874 | | 0.039034412 | down | -0.65044366 | 0.001255801 | down | 1.21214193 | 0.191457117 | up |
| SLCO1A2 | -1.752501096 | | 9.89E-09 | down | -1.492947567 | | 0.046549819 | down | -0.855817112 | 0.001551079 | down | 0.677997328 | 0.567294455 | up |
| PRB4 | -5.148198607 | | 0.000000787 | down | -2.211287043 | | 0.02801553 | down | -2.349114631 | 0.001731091 | down | 0.312141756 | 0.481195694 | up |
| ODAM | -3.703027533 | | 0.00000778 | down | -1.078794836 | | 0.043741859 | down | -0.506850229 | 0.003805066 | down | 3.261271681 | 0.282853417 | up |
| TSPAN8 | -1.834081884 | | 1.04E-08 | down | -1.179495862 | | 0.029588741 | down | -1.032586507 | 0.009740632 | down | -0.084181774 | 0.943018794 | down |
| HMGCS2 | -3.922557192 | | 6.01E-11 | down | -1.471873861 | | 0.029359979 | down | -1.798143684 | 0.010178476 | down | -0.26356532 | 0.614575293 | down |
| GP2 | -2.353901168 | | 0.000584846 | down | -1.99334482 | | 0.041055631 | down | -1.449081996 | 0.022230126 | down | - | - | - |
| SLC26A4 | 1.509434774 | | 0.001417395 | up | 1.508947118 | | 0.044638632 | up | 1.663495729 | 0.028005944 | up | 1.972105294 | 0.34955891 | up |
| Log_2_FC, log_2_ fold change | | | |  |  |  |  |  |  |  |  |  |  |  |
| adj. p-value, adjusted p-value | | | |  |  |  |  |  |  |  |  |  |  |  |

| **Table S2.** GO functional annotation enrichment analysis of upregulated co-DEGs in CRSwNP | | | | | | |  |
| --- | --- | --- | --- | --- | --- | --- | --- |
| **Ontology** | **ID** | **Description** | | **adj. p-value** | **Count** | **Gene ID** | |
| BP | GO:0002697 | regulation of immune effector process | | 2.39E-07 | 12 | SERPINB4/CLC/ITGAM/IL2RA/CFI/BTK/C1QB/ITGB2/FCER1G/CD300A/HMOX1/LILRB1 | |
| BP | GO:0050900 | leukocyte migration | | 2.39E-07 | 12 | CCL18/CCL13/ITGAM/HCK/PLA2G7/SELPLG/ITGB2/FCER1G/CCL8/HRH1/CD300A/HMOX1 | |
| BP | GO:0002703 | regulation of leukocyte mediated immunity | | 2.39E-07 | 9 | SERPINB4/CLC/ITGAM/BTK/ITGB2/FCER1G/CD300A/HMOX1/LILRB1 | |
| BP | GO:0002886 | regulation of myeloid leukocyte mediated immunity | | 5.66E-07 | 6 | ITGAM/BTK/ITGB2/FCER1G/CD300A/HMOX1 | |
| BP | GO:0097529 | myeloid leukocyte migration | | 4.06E-06 | 8 | CCL18/CCL13/PLA2G7/ITGB2/FCER1G/CCL8/HRH1/CD300A | |
| BP | GO:0097530 | granulocyte migration | | 4.79E-06 | 7 | CCL18/CCL13/ITGB2/FCER1G/CCL8/HRH1/CD300A | |
| BP | GO:0050727 | regulation of inflammatory response | | 6.36E-06 | 10 | CCL18/ITGAM/HCK/IL2RA/CFI/PLA2G7/BTK/C1QB/ITGB2/FCER1G | |
| BP | GO:0043300 | regulation of leukocyte degranulation | | 6.43E-06 | 5 | ITGAM/ITGB2/FCER1G/CD300A/HMOX1 | |
| BP | GO:0002683 | negative regulation of immune system process | | 6.43E-06 | 10 | SERPINB4/IL2RA/BTK/SAMSN1/CD300 LF/FCER1G/CD300A/HMOX1/LILRB1/TMEM176A | |
| BP | GO:0002695 | negative regulation of leukocyte activation | | 1.16E-05 | 7 | IL2RA/BTK/SAMSN1/CD300 LF/CD300A/HMOX1/LILRB1 | |
| BP | GO:0007229 | integrin-mediated signaling pathway | | 1.18E-05 | 6 | ITGAM/HCK/PLEK/ITGB2/FCER1G/TYROBP | |
| BP | GO:0045088 | regulation of innate immune response | | 1.32E-05 | 9 | SERPINB4/ITGAM/HCK/BTK/ITGB2/CD300 LF/FCER1G/CD300A/LILRB1 | |
| BP | GO:0045576 | mast cell activation | | 1.81E-05 | 5 | BTK/CD300 LF/FCER1G/CD300A/HMOX1 | |
| BP | GO:0050866 | negative regulation of cell activation | | 1.81E-05 | 7 | IL2RA/BTK/SAMSN1/CD300 LF/CD300A/HMOX1/LILRB1 | |
| BP | GO:0070661 | leukocyte proliferation | | 2.05E-05 | 8 | CLC/CD180/IL2RA/BTK/CCL8/CD4/CD300A/LILRB1 | |
| BP | GO:0072677 | eosinophil migration | | 2.39E-05 | 4 | CCL13/CCL8/HRH1/CD300A | |
| BP | GO:0071621 | granulocyte chemotaxis | | 2.40E-05 | 6 | CCL18/CCL13/ITGB2/FCER1G/CCL8/HRH1 | |
| BP | GO:0031349 | positive regulation of defense response | | 3.08E-05 | 9 | CCL18/ITGAM/HCK/PLA2G7/BTK/ITGB2/CD300 LF/FCER1G/CD300A | |
| BP | GO:0002699 | positive regulation of immune effector process | | 3.08E-05 | 7 | ITGAM/BTK/ITGB2/FCER1G/CD300A/HMOX1/LILRB1 | |
| BP | GO:0002888 | positive regulation of myeloid leukocyte mediated immunity | | 3.08E-05 | 4 | ITGAM/BTK/ITGB2/FCER1G | |
| BP | GO:0050777 | negative regulation of immune response | | 3.23E-05 | 6 | SERPINB4/IL2RA/SAMSN1/CD300A/HMOX1/LILRB1 | |
| BP | GO:0050729 | positive regulation of inflammatory response | | 3.23E-05 | 6 | CCL18/ITGAM/PLA2G7/BTK/ITGB2/FCER1G | |
| BP | GO:0070663 | regulation of leukocyte proliferation | | 3.23E-05 | 7 | CLC/IL2RA/BTK/CCL8/CD4/CD300A/LILRB1 | |
| BP | GO:0030595 | leukocyte chemotaxis | | 3.23E-05 | 7 | CCL18/CCL13/PLA2G7/ITGB2/FCER1G/CCL8/HRH1 | |
| BP | GO:0042554 | superoxide anion generation | | 3.77E-05 | 4 | NCF2/ITGAM/ITGB2/CYBB | |
| BP | GO:0070664 | negative regulation of leukocyte proliferation | | 3.89E-05 | 5 | IL2RA/BTK/CCL8/CD300A/LILRB1 | |
| BP | GO:0002718 | regulation of cytokine production involved in immune response | | 4.28E-05 | 5 | CLC/BTK/FCER1G/HMOX1/LILRB1 | |
| BP | GO:0002758 | innate immune response-activating signal transduction | | 4.46E-05 | 7 | ITGAM/HCK/BTK/ITGB2/CD300 LF/FCER1G/CD300A | |
| BP | GO:0050764 | regulation of phagocytosis | | 4.82E-05 | 5 | ITGAM/HCK/CD300 LF/FCER1G/CD300A | |
| BP | GO:0043312 | neutrophil degranulation | | 5.73E-05 | 9 | FPR1/ITGAM/HK3/ITGB2/FCER1G/RNASE2/TYROBP/CD300A/CYBB | |
| BP | GO:0002283 | neutrophil activation involved in immune response | | 5.84E-05 | 9 | FPR1/ITGAM/HK3/ITGB2/FCER1G/RNASE2/TYROBP/CD300A/CYBB | |
| BP | GO:0002819 | regulation of adaptive immune response | | 6.18E-05 | 6 | CLC/BTK/SAMSN1/FCER1G/CD4/LILRB1 | |
| BP | GO:0002218 | activation of innate immune response | | 6.21E-05 | 7 | ITGAM/HCK/BTK/ITGB2/CD300 LF/FCER1G/CD300A | |
| BP | GO:0042119 | neutrophil activation | | 6.21E-05 | 9 | FPR1/ITGAM/HK3/ITGB2/FCER1G/RNASE2/TYROBP/CD300A/CYBB | |
| BP | GO:0002446 | neutrophil mediated immunity | | 6.21E-05 | 9 | FPR1/ITGAM/HK3/ITGB2/FCER1G/RNASE2/TYROBP/CD300A/CYBB | |
| BP | GO:1902563 | regulation of neutrophil activation | | 6.55E-05 | 3 | ITGAM/ITGB2/CD300A | |
| BP | GO:0033003 | regulation of mast cell activation | | 6.57E-05 | 4 | CD300 LF/FCER1G/CD300A/HMOX1 | |
| BP | GO:0046651 | lymphocyte proliferation | | 7.55E-05 | 7 | CLC/CD180/IL2RA/BTK/CD4/CD300A/LILRB1 | |
| BP | GO:0032943 | mononuclear cell proliferation | | 7.63E-05 | 7 | CLC/CD180/IL2RA/BTK/CD4/CD300A/LILRB1 | |
| BP | GO:0050867 | positive regulation of cell activation | | 7.63E-05 | 8 | ITGAM/IL2RA/BTK/PLEK/ITGB2/FCER1G/CD4/LILRB1 | |
| BP | GO:0033004 | negative regulation of mast cell activation | | 7.66E-05 | 3 | CD300 LF/CD300A/HMOX1 | |
| BP | GO:0002367 | cytokine production involved in immune response | | 7.82E-05 | 5 | CLC/BTK/FCER1G/HMOX1/LILRB1 | |
| BP | GO:0043303 | mast cell degranulation | | 9.25E-05 | 4 | BTK/FCER1G/CD300A/HMOX1 | |
| BP | GO:0002279 | mast cell activation involved in immune response | | 9.65E-05 | 4 | BTK/FCER1G/CD300A/HMOX1 | |
| BP | GO:0002704 | negative regulation of leukocyte mediated immunity | | 9.65E-05 | 4 | SERPINB4/CD300A/HMOX1/LILRB1 | |
| BP | GO:0030593 | neutrophil chemotaxis | | 9.65E-05 | 5 | CCL18/CCL13/ITGB2/FCER1G/CCL8 | |
| BP | GO:0002448 | mast cell mediated immunity | | 0.0001 | 4 | BTK/FCER1G/CD300A/HMOX1 | |
| BP | GO:0071887 | leukocyte apoptotic process | | 0.00012 | 5 | CLC/IL2RA/BTK/FCER1G/LILRB1 | |
| BP | GO:0060326 | cell chemotaxis | | 0.00012 | 7 | CCL18/CCL13/PLA2G7/ITGB2/FCER1G/CCL8/HRH1 | |
| BP | GO:0032103 | positive regulation of response to external stimulus | | 0.00012 | 7 | CCL18/ITGAM/CD180/PLA2G7/BTK/ITGB2/FCER1G | |
| BP | GO:0002698 | negative regulation of immune effector process | | 0.00014 | 5 | SERPINB4/IL2RA/CD300A/HMOX1/LILRB1 | |
| BP | GO:1990266 | neutrophil migration | | 0.00014 | 5 | CCL18/CCL13/ITGB2/FCER1G/CCL8 | |
| BP | GO:0045089 | positive regulation of innate immune response | | 0.00015 | 7 | ITGAM/HCK/BTK/ITGB2/CD300 LF/FCER1G/CD300A | |
| BP | GO:0002768 | immune response-regulating cell surface receptor signaling pathway | | 0.00017 | 8 | FPR1/HCK/BTK/MS4A2/FCER1G/CD4/CD300A/LILRB1 | |
| BP | GO:0050670 | regulation of lymphocyte proliferation | | 0.00017 | 6 | CLC/IL2RA/BTK/CD4/CD300A/LILRB1 | |
| BP | GO:0032944 | regulation of mononuclear cell proliferation | | 0.00017 | 6 | CLC/IL2RA/BTK/CD4/CD300A/LILRB1 | |
| BP | GO:0006909 | phagocytosis | | 0.00027 | 7 | NCF2/ITGAM/HCK/ITGB2/CD300 LF/FCER1G/CD300A | |
| BP | GO:0002224 | toll-like receptor signaling pathway | | 0.00027 | 5 | ITGAM/BTK/ITGB2/CD300 LF/CD300A | |
| BP | GO:0002449 | lymphocyte mediated immunity | | 0.00028 | 7 | SERPINB4/CLC/CFI/BTK/C1QB/FCER1G/LILRB1 | |
| BP | GO:0002700 | regulation of production of molecular mediator of immune response | | 0.00028 | 5 | CLC/BTK/FCER1G/HMOX1/LILRB1 | |
| BP | GO:0002548 | monocyte chemotaxis | | 0.00029 | 4 | CCL18/CCL13/PLA2G7/CCL8 | |
| BP | GO:0043302 | positive regulation of leukocyte degranulation | | 0.0003 | 3 | ITGAM/ITGB2/FCER1G | |
| BP | GO:0048245 | eosinophil chemotaxis | | 0.0003 | 3 | CCL13/CCL8/HRH1 | |
| BP | GO:0032418 | lysosome localization | | 0.0003 | 4 | BTK/FCER1G/CD300A/HMOX1 | |
| BP | GO:0002460 | adaptive immune response based on somatic recombination of immune receptors built from immunoglobulin superfamily domains | | 0.0003 | 7 | CLC/CFI/BTK/C1QB/FCER1G/CD4/LILRB1 | |
| BP | GO:0051250 | negative regulation of lymphocyte activation | | 0.0003 | 5 | IL2RA/BTK/SAMSN1/CD300A/LILRB1 | |
| BP | GO:0060627 | regulation of vesicle-mediated transport | | 0.00031 | 8 | ITGAM/HCK/ITGB2/CD300 LF/FCER1G/CD300A/HMOX1/LILRB1 | |
| BP | GO:0006801 | superoxide metabolic process | | 0.00036 | 4 | NCF2/ITGAM/ITGB2/CYBB | |
| BP | GO:0002822 | regulation of adaptive immune response based on somatic recombination of immune receptors built from immunoglobulin superfamily domains | | 0.00036 | 5 | CLC/BTK/FCER1G/CD4/LILRB1 | |
| BP | GO:0032945 | negative regulation of mononuclear cell proliferation | | 0.00036 | 4 | IL2RA/BTK/CD300A/LILRB1 | |
| BP | GO:0050672 | negative regulation of lymphocyte proliferation | | 0.00036 | 4 | IL2RA/BTK/CD300A/LILRB1 | |
| BP | GO:0002706 | regulation of lymphocyte mediated immunity | | 0.00037 | 5 | SERPINB4/CLC/BTK/FCER1G/LILRB1 | |
| BP | GO:0002696 | positive regulation of leukocyte activation | | 0.00037 | 7 | ITGAM/IL2RA/BTK/ITGB2/FCER1G/CD4/LILRB1 | |
| BP | GO:1903305 | regulation of regulated secretory pathway | | 0.00039 | 5 | ITGAM/ITGB2/FCER1G/CD300A/HMOX1 | |
| BP | GO:0018108 | peptidyl-tyrosine phosphorylation | | 0.00046 | 7 | HCK/BTK/SAMSN1/ITGB2/CD4/CD300A/CSF1R | |
| BP | GO:0030100 | regulation of endocytosis | | 0.00047 | 6 | ITGAM/HCK/CD300 LF/FCER1G/CD300A/LILRB1 | |
| BP | GO:0018212 | peptidyl-tyrosine modification | | 0.00047 | 7 | HCK/BTK/SAMSN1/ITGB2/CD4/CD300A/CSF1R | |
| BP | GO:1902105 | regulation of leukocyte differentiation | | 0.00051 | 6 | IL2RA/BTK/TYROBP/CD4/LILRB1/TMEM176A | |
| BP | GO:0043304 | regulation of mast cell degranulation | | 0.00058 | 3 | FCER1G/CD300A/HMOX1 | |
| BP | GO:0033006 | regulation of mast cell activation involved in immune response | | 0.00063 | 3 | FCER1G/CD300A/HMOX1 | |
| BP | GO:0050869 | negative regulation of B cell activation | | 0.00063 | 3 | BTK/SAMSN1/CD300A | |
| BP | GO:0071674 | mononuclear cell migration | | 0.00067 | 4 | CCL18/CCL13/PLA2G7/CCL8 | |
| BP | GO:0002221 | pattern recognition receptor signaling pathway | | 0.00086 | 5 | ITGAM/BTK/ITGB2/CD300 LF/CD300A | |
| BP | GO:0045730 | respiratory burst | | 0.0009 | 3 | NCF2/HCK/CYBB | |
| BP | GO:0042035 | regulation of cytokine biosynthetic process | | 0.00097 | 4 | CD4/HMOX1/CYBB/LILRB1 | |
| BP | GO:0030316 | osteoclast differentiation | | 0.00099 | 4 | FCER1G/TYROBP/CSF1R/LILRB1 | |
| BP | GO:0002755 | MyD88-dependent toll-like receptor signaling pathway | | 0.00113 | 3 | BTK/CD300 LF/CD300A | |
| BP | GO:0002573 | myeloid leukocyte differentiation | | 0.00127 | 5 | FCER1G/TYROBP/CD4/CSF1R/LILRB1 | |
| BP | GO:0051249 | regulation of lymphocyte activation | | 0.00136 | 7 | CLC/IL2RA/BTK/SAMSN1/CD4/CD300A/LILRB1 | |
| BP | GO:0042089 | cytokine biosynthetic process | | 0.00139 | 4 | CD4/HMOX1/CYBB/LILRB1 | |
| BP | GO:0007159 | leukocyte cell-cell adhesion | | 0.00139 | 6 | IL2RA/SELPLG/ITGB2/CD4/CD300A/LILRB1 | |
| BP | GO:0042107 | cytokine metabolic process | | 0.00142 | 4 | CD4/HMOX1/CYBB/LILRB1 | |
| BP | GO:0017157 | regulation of exocytosis | | 0.00142 | 5 | ITGAM/ITGB2/FCER1G/CD300A/HMOX1 | |
| BP | GO:0002437 | inflammatory response to antigenic stimulus | | 0.00166 | 3 | IL2RA/BTK/FCER1G | |
| BP | GO:0006911 | phagocytosis, engulfment | | 0.00171 | 4 | ITGAM/ITGB2/FCER1G/CD300A | |
| BP | GO:0099024 | plasma membrane invagination | | 0.00228 | 4 | ITGAM/ITGB2/FCER1G/CD300A | |
| BP | GO:1903307 | positive regulation of regulated secretory pathway | | 0.00271 | 3 | ITGAM/ITGB2/FCER1G | |
| BP | GO:0010324 | membrane invagination | | 0.00282 | 4 | ITGAM/ITGB2/FCER1G/CD300A | |
| BP | GO:0001820 | serotonin secretion | | 0.00282 | 2 | FCER1G/LILRB1 | |
| BP | GO:0002664 | regulation of T cell tolerance induction | | 0.00282 | 2 | CLC/IL2RA | |
| BP | GO:0002863 | positive regulation of inflammatory response to antigenic stimulus | | 0.00282 | 2 | BTK/FCER1G | |
| BP | GO:0002887 | negative regulation of myeloid leukocyte mediated immunity | | 0.00282 | 2 | CD300A/HMOX1 | |
| BP | GO:2001198 | regulation of dendritic cell differentiation | | 0.00282 | 2 | LILRB1/TMEM176A | |
| BP | GO:0002705 | positive regulation of leukocyte mediated immunity | | 0.00285 | 4 | ITGAM/BTK/ITGB2/FCER1G | |
| BP | GO:0050730 | regulation of peptidyl-tyrosine phosphorylation | | 0.00292 | 5 | SAMSN1/ITGB2/CD4/CD300A/CSF1R | |
| BP | GO:0070374 | positive regulation of ERK1 and ERK2 cascade | | 0.00292 | 5 | CCL18/CCL13/CCL8/CD4/CSF1R | |
| BP | GO:0043301 | negative regulation of leukocyte degranulation | | 0.00328 | 2 | CD300A/HMOX1 | |
| BP | GO:0045953 | negative regulation of natural killer cell mediated cytotoxicity | | 0.00328 | 2 | SERPINB4/LILRB1 | |
| BP | GO:0045806 | negative regulation of endocytosis | | 0.00338 | 3 | CD300 LF/CD300A/LILRB1 | |
| BP | GO:0042108 | positive regulation of cytokine biosynthetic process | | 0.0035 | 3 | CD4/HMOX1/CYBB | |
| BP | GO:0050766 | positive regulation of phagocytosis | | 0.0035 | 3 | ITGAM/CD300 LF/FCER1G | |
| BP | GO:0002517 | T cell tolerance induction | | 0.00369 | 2 | CLC/IL2RA | |
| BP | GO:0002716 | negative regulation of natural killer cell mediated immunity | | 0.00369 | 2 | SERPINB4/LILRB1 | |
| BP | GO:0002864 | regulation of acute inflammatory response to antigenic stimulus | | 0.00369 | 2 | BTK/FCER1G | |
| BP | GO:2001204 | regulation of osteoclast development | | 0.00369 | 2 | TYROBP/LILRB1 | |
| BP | GO:0002429 | immune response-activating cell surface receptor signaling pathway | | 0.00391 | 6 | FPR1/HCK/BTK/FCER1G/CD4/CD300A | |
| BP | GO:0002440 | production of molecular mediator of immune response | | 0.004 | 5 | CLC/BTK/FCER1G/HMOX1/LILRB1 | |
| BP | GO:1903706 | regulation of hemopoiesis | | 0.00405 | 6 | IL2RA/BTK/TYROBP/CD4/LILRB1/TMEM176A | |
| BP | GO:0002673 | regulation of acute inflammatory response | | 0.00407 | 4 | CFI/BTK/C1QB/FCER1G | |
| BP | GO:0060099 | regulation of phagocytosis, engulfment | | 0.00418 | 2 | ITGAM/CD300A | |
| BP | GO:0008360 | regulation of cell shape | | 0.00421 | 4 | CCL13/HCK/ITGB2/CSF1R | |
| BP | GO:0048247 | lymphocyte chemotaxis | | 0.00428 | 3 | CCL18/CCL13/CCL8 | |
| BP | GO:0042129 | regulation of T cell proliferation | | 0.00435 | 4 | CLC/IL2RA/CD4/LILRB1 | |
| BP | GO:0150078 | positive regulation of neuroinflammatory response | | 0.00467 | 2 | ITGAM/ITGB2 | |
| BP | GO:1905153 | regulation of membrane invagination | | 0.00467 | 2 | ITGAM/CD300A | |
| BP | GO:0031670 | cellular response to nutrient | | 0.00519 | 3 | FOLR2/HMOX1/CYBB | |
| BP | GO:0001911 | negative regulation of leukocyte mediated cytotoxicity | | 0.00522 | 2 | SERPINB4/LILRB1 | |
| BP | GO:0006837 | serotonin transport | | 0.00522 | 2 | FCER1G/LILRB1 | |
| BP | GO:0030889 | negative regulation of B cell proliferation | | 0.00522 | 2 | BTK/CD300A | |
| BP | GO:0042110 | T cell activation | | 0.00549 | 6 | CLC/IL2RA/FCER1G/CD4/CD300A/LILRB1 | |
| BP | GO:0010919 | regulation of inositol phosphate biosynthetic process | | 0.00581 | 2 | PLEK/HRH1 | |
| BP | GO:0032930 | positive regulation of superoxide anion generation | | 0.00581 | 2 | ITGAM/ITGB2 | |
| BP | GO:0070227 | lymphocyte apoptotic process | | 0.00581 | 3 | CLC/IL2RA/BTK | |
| BP | GO:0050863 | regulation of T cell activation | | 0.00628 | 5 | CLC/IL2RA/CD4/CD300A/LILRB1 | |
| BP | GO:0002643 | regulation of tolerance induction | | 0.00639 | 2 | CLC/IL2RA | |
| BP | GO:1903978 | regulation of microglial cell activation | | 0.00639 | 2 | ITGAM/ITGB2 | |
| BP | GO:0042098 | T cell proliferation | | 0.00685 | 4 | CLC/IL2RA/CD4/LILRB1 | |
| BP | GO:0071346 | cellular response to interferon-gamma | | 0.00685 | 4 | CCL18/CCL13/HCK/CCL8 | |
| BP | GO:0031342 | negative regulation of cell killing | | 0.00697 | 2 | SERPINB4/LILRB1 | |
| BP | GO:0050765 | negative regulation of phagocytosis | | 0.00697 | 2 | CD300 LF/CD300A | |
| BP | GO:0002685 | regulation of leukocyte migration | | 0.00699 | 4 | PLA2G7/CCL8/CD300A/HMOX1 | |
| BP | GO:0042116 | macrophage activation | | 0.0074 | 3 | ITGAM/ITGB2/TYROBP | |
| BP | GO:0044106 | cellular amine metabolic process | | 0.00757 | 3 | TDO2/ITGAM/ITGB2 | |
| BP | GO:0036035 | osteoclast development | | 0.00757 | 2 | TYROBP/LILRB1 | |
| BP | GO:0070372 | regulation of ERK1 and ERK2 cascade | | 0.00761 | 5 | CCL18/CCL13/CCL8/CD4/CSF1R | |
| BP | GO:0140029 | exocytic process | | 0.00773 | 3 | BTK/PLEK/FCER1G | |
| BP | GO:0038093 | Fc receptor signaling pathway | | 0.00773 | 4 | HCK/BTK/MS4A2/FCER1G | |
| BP | GO:0045921 | positive regulation of exocytosis | | 0.00785 | 3 | ITGAM/ITGB2/FCER1G | |
| BP | GO:0032928 | regulation of superoxide anion generation | | 0.00785 | 2 | ITGAM/ITGB2 | |
| BP | GO:0042053 | regulation of dopamine metabolic process | | 0.00785 | 2 | ITGAM/ITGB2 | |
| BP | GO:0042069 | regulation of catecholamine metabolic process | | 0.00785 | 2 | ITGAM/ITGB2 | |
| BP | GO:0042533 | tumor necrosis factor biosynthetic process | | 0.00785 | 2 | CYBB/LILRB1 | |
| BP | GO:0042534 | regulation of tumor necrosis factor biosynthetic process | | 0.00785 | 2 | CYBB/LILRB1 | |
| BP | GO:0097242 | amyloid-beta clearance | | 0.00785 | 2 | ITGAM/ITGB2 | |
| BP | GO:0002438 | acute inflammatory response to antigenic stimulus | | 0.00848 | 2 | BTK/FCER1G | |
| BP | GO:0002861 | regulation of inflammatory response to antigenic stimulus | | 0.00848 | 2 | BTK/FCER1G | |
| BP | GO:0070098 | chemokine-mediated signaling pathway | | 0.00848 | 3 | CCL18/CCL13/CCL8 | |
| BP | GO:2000106 | regulation of leukocyte apoptotic process | | 0.00848 | 3 | BTK/FCER1G/LILRB1 | |
| BP | GO:0070371 | ERK1 and ERK2 cascade | | 0.00872 | 5 | CCL18/CCL13/CCL8/CD4/CSF1R | |
| BP | GO:0034341 | response to interferon-gamma | | 0.00877 | 4 | CCL18/CCL13/HCK/CCL8 | |
| BP | GO:0009308 | amine metabolic process | | 0.00913 | 3 | TDO2/ITGAM/ITGB2 | |
| BP | GO:0006898 | receptor-mediated endocytosis | | 0.00913 | 5 | CFI/FOLR2/ITGB2/FCER1G/LILRB1 | |
| BP | GO:0042100 | B cell proliferation | | 0.00936 | 3 | CD180/BTK/CD300A | |
| BP | GO:0071404 | cellular response to low-density lipoprotein particle stimulus | | 0.00971 | 2 | ITGB2/FCER1G | |
| BP | GO:1903306 | negative regulation of regulated secretory pathway | | 0.00971 | 2 | CD300A/HMOX1 | |
| BP | GO:1990868 | response to chemokine | | 0.01033 | 3 | CCL18/CCL13/CCL8 | |
| BP | GO:1990869 | cellular response to chemokine | | 0.01033 | 3 | CCL18/CCL13/CCL8 | |
| BP | GO:0002719 | negative regulation of cytokine production involved in immune response | | 0.01033 | 2 | HMOX1/LILRB1 | |
| BP | GO:1903010 | regulation of bone development | | 0.01033 | 2 | TYROBP/LILRB1 | |
| BP | GO:0002526 | acute inflammatory response | | 0.01041 | 4 | CFI/BTK/C1QB/FCER1G | |
| BP | GO:0031532 | actin cytoskeleton reorganization | | 0.01041 | 3 | HCK/PLEK/CSF1R | |
| BP | GO:0016064 | immunoglobulin mediated immune response | | 0.0105 | 4 | CFI/BTK/C1QB/FCER1G | |
| BP | GO:0019724 | B cell mediated immunity | | 0.01062 | 4 | CFI/BTK/C1QB/FCER1G | |
| BP | GO:0002507 | tolerance induction | | 0.01082 | 2 | CLC/IL2RA | |
| BP | GO:0150077 | regulation of neuroinflammatory response | | 0.01082 | 2 | ITGAM/ITGB2 | |
| BP | GO:0007584 | response to nutrient | | 0.01099 | 4 | FOLR2/CD4/HMOX1/CYBB | |
| BP | GO:0002824 | positive regulation of adaptive immune response based on somatic recombination of immune receptors built from immunoglobulin superfamily domains | | 0.01131 | 3 | BTK/FCER1G/CD4 | |
| BP | GO:0043032 | positive regulation of macrophage activation | | 0.01151 | 2 | ITGAM/ITGB2 | |
| BP | GO:0002821 | positive regulation of adaptive immune response | | 0.01279 | 3 | BTK/FCER1G/CD4 | |
| BP | GO:0072676 | lymphocyte migration | | 0.01279 | 3 | CCL18/CCL13/CCL8 | |
| BP | GO:0050868 | negative regulation of T cell activation | | 0.01292 | 3 | IL2RA/CD300A/LILRB1 | |
| BP | GO:0002675 | positive regulation of acute inflammatory response | | 0.01292 | 2 | BTK/FCER1G | |
| BP | GO:0032958 | inositol phosphate biosynthetic process | | 0.01292 | 2 | PLEK/HRH1 | |
| BP | GO:0061082 | myeloid leukocyte cytokine production | | 0.01292 | 2 | FCER1G/HMOX1 | |
| BP | GO:0071216 | cellular response to biotic stimulus | | 0.01294 | 4 | CD180/HCK/BTK/LILRB1 | |
| BP | GO:0031623 | receptor internalization | | 0.01306 | 3 | ITGB2/FCER1G/LILRB1 | |
| BP | GO:0050690 | regulation of defense response to virus by virus | | 0.01363 | 2 | HCK/CD4 | |
| BP | GO:0015696 | ammonium transport | | 0.01363 | 3 | BTK/FCER1G/LILRB1 | |
| BP | GO:0010543 | regulation of platelet activation | | 0.01435 | 2 | PLEK/FCER1G | |
| BP | GO:0090314 | positive regulation of protein targeting to membrane | | 0.01435 | 2 | ITGAM/ITGB2 | |
| BP | GO:0055094 | response to lipoprotein particle | | 0.01516 | 2 | ITGB2/FCER1G | |
| BP | GO:0002761 | regulation of myeloid leukocyte differentiation | | 0.01516 | 3 | TYROBP/CD4/LILRB1 | |
| BP | GO:0030099 | myeloid cell differentiation | | 0.01529 | 5 | FCER1G/TYROBP/CD4/CSF1R/LILRB1 | |
| BP | GO:0045920 | negative regulation of exocytosis | | 0.01598 | 2 | CD300A/HMOX1 | |
| BP | GO:0038095 | Fc-epsilon receptor signaling pathway | | 0.01607 | 3 | BTK/MS4A2/FCER1G | |
| BP | GO:0001774 | microglial cell activation | | 0.01648 | 2 | ITGAM/ITGB2 | |
| BP | GO:0002269 | leukocyte activation involved in inflammatory response | | 0.01648 | 2 | ITGAM/ITGB2 | |
| BP | GO:0034142 | toll-like receptor 4 signaling pathway | | 0.01648 | 2 | ITGAM/ITGB2 | |
| BP | GO:0071402 | cellular response to lipoprotein particle stimulus | | 0.01648 | 2 | ITGB2/FCER1G | |
| BP | GO:0098751 | bone cell development | | 0.01648 | 2 | TYROBP/LILRB1 | |
| BP | GO:0002701 | negative regulation of production of molecular mediator of immune response | | 0.01688 | 2 | HMOX1/LILRB1 | |
| BP | GO:0010922 | positive regulation of phosphatase activity | | 0.01688 | 2 | PLEK/CD300A | |
| BP | GO:0030224 | monocyte differentiation | | 0.01688 | 2 | CD4/CSF1R | |
| BP | GO:0090322 | regulation of superoxide metabolic process | | 0.01688 | 2 | ITGAM/ITGB2 | |
| BP | GO:1903131 | mononuclear cell differentiation | | 0.01688 | 2 | CD4/CSF1R | |
| BP | GO:2000249 | regulation of actin cytoskeleton reorganization | | 0.01688 | 2 | HCK/CSF1R | |
| BP | GO:0001819 | positive regulation of cytokine production | | 0.01688 | 5 | FCER1G/CD4/CSF1R/HMOX1/CYBB | |
| BP | GO:0032386 | regulation of intracellular transport | | 0.01688 | 5 | ITGAM/ITGB2/FCER1G/CD300A/HMOX1 | |
| BP | GO:1903038 | negative regulation of leukocyte cell-cell adhesion | | 0.01688 | 3 | IL2RA/CD300A/LILRB1 | |
| BP | GO:0002707 | negative regulation of lymphocyte mediated immunity | | 0.01763 | 2 | SERPINB4/LILRB1 | |
| BP | GO:0090313 | regulation of protein targeting to membrane | | 0.01855 | 2 | ITGAM/ITGB2 | |
| BP | GO:0032680 | regulation of tumor necrosis factor production | | 0.01864 | 3 | FCER1G/CYBB/LILRB1 | |
| BP | GO:0002576 | platelet degranulation | | 0.01897 | 3 | F13A1/PLEK/FCER1G | |
| BP | GO:0032640 | tumor necrosis factor production | | 0.01904 | 3 | FCER1G/CYBB/LILRB1 | |
| BP | GO:0038083 | peptidyl-tyrosine autophosphorylation | | 0.01904 | 2 | HCK/BTK | |
| BP | GO:0046636 | negative regulation of alpha-beta T cell activation | | 0.01904 | 2 | CD300A/LILRB1 | |
| BP | GO:0071276 | cellular response to cadmium ion | | 0.01904 | 2 | HMOX1/CYBB | |
| BP | GO:0019932 | second-messenger-mediated signaling | | 0.01916 | 5 | FPR1/BTK/PLEK/HRH1/CD4 | |
| BP | GO:0002831 | regulation of response to biotic stimulus | | 0.01918 | 3 | CD180/IL2RA/LILRB1 | |
| BP | GO:0071347 | cellular response to interleukin-1 | | 0.01918 | 3 | CCL18/CCL13/CCL8 | |
| BP | GO:1903555 | regulation of tumor necrosis factor superfamily cytokine production | | 0.01944 | 3 | FCER1G/CYBB/LILRB1 | |
| BP | GO:0002714 | positive regulation of B cell mediated immunity | | 0.01944 | 2 | BTK/FCER1G | |
| BP | GO:0002891 | positive regulation of immunoglobulin mediated immune response | | 0.01944 | 2 | BTK/FCER1G | |
| BP | GO:0033238 | regulation of cellular amine metabolic process | | 0.01944 | 2 | ITGAM/ITGB2 | |
| BP | GO:0042417 | dopamine metabolic process | | 0.02037 | 2 | ITGAM/ITGB2 | |
| BP | GO:0072593 | reactive oxygen species metabolic process | | 0.02067 | 4 | NCF2/ITGAM/ITGB2/CYBB | |
| BP | GO:0071706 | tumor necrosis factor superfamily cytokine production | | 0.02067 | 3 | FCER1G/CYBB/LILRB1 | |
| BP | GO:0002820 | negative regulation of adaptive immune response | | 0.02077 | 2 | SAMSN1/LILRB1 | |
| BP | GO:0034122 | negative regulation of toll-like receptor signaling pathway | | 0.02077 | 2 | CD300 LF/CD300A | |
| BP | GO:0046006 | regulation of activated T cell proliferation | | 0.02077 | 2 | CLC/IL2RA | |
| BP | GO:0061900 | glial cell activation | | 0.02077 | 2 | ITGAM/ITGB2 | |
| BP | GO:0097028 | dendritic cell differentiation | | 0.02077 | 2 | LILRB1/TMEM176A | |
| BP | GO:0045807 | positive regulation of endocytosis | | 0.02266 | 3 | ITGAM/CD300 LF/FCER1G | |
| BP | GO:0042269 | regulation of natural killer cell mediated cytotoxicity | | 0.02266 | 2 | SERPINB4/LILRB1 | |
| BP | GO:1902107 | positive regulation of leukocyte differentiation | | 0.023 | 3 | IL2RA/BTK/CD4 | |
| BP | GO:1903037 | regulation of leukocyte cell-cell adhesion | | 0.02441 | 4 | IL2RA/CD4/CD300A/LILRB1 | |
| BP | GO:0002715 | regulation of natural killer cell mediated immunity | | 0.02441 | 2 | SERPINB4/LILRB1 | |
| BP | GO:0050798 | activated T cell proliferation | | 0.02441 | 2 | CLC/IL2RA | |
| BP | GO:0002686 | negative regulation of leukocyte migration | | 0.02529 | 2 | CD300A/HMOX1 | |
| BP | GO:0032653 | regulation of interleukin-10 production | | 0.02529 | 2 | FCER1G/LILRB1 | |
| BP | GO:0050852 | T cell receptor signaling pathway | | 0.02613 | 3 | BTK/CD4/CD300A | |
| BP | GO:0045824 | negative regulation of innate immune response | | 0.02618 | 2 | SERPINB4/LILRB1 | |
| BP | GO:0042113 | B cell activation | | 0.02678 | 4 | CD180/BTK/SAMSN1/CD300A | |
| BP | GO:0070555 | response to interleukin-1 | | 0.02678 | 3 | CCL18/CCL13/CCL8 | |
| BP | GO:0032613 | interleukin-10 production | | 0.02686 | 2 | FCER1G/LILRB1 | |
| BP | GO:0150076 | neuroinflammatory response | | 0.02686 | 2 | ITGAM/ITGB2 | |
| BP | GO:0050732 | negative regulation of peptidyl-tyrosine phosphorylation | | 0.02902 | 2 | SAMSN1/CD300A | |
| BP | GO:0001906 | cell killing | | 0.02933 | 3 | SERPINB4/CCL13/LILRB1 | |
| BP | GO:0070231 | T cell apoptotic process | | 0.02991 | 2 | CLC/IL2RA | |
| BP | GO:0009615 | response to virus | | 0.02991 | 4 | IL2RA/CCL8/RNASE2/LILRB1 | |
| BP | GO:2000107 | negative regulation of leukocyte apoptotic process | | 0.03087 | 2 | FCER1G/LILRB1 | |
| BP | GO:0002712 | regulation of B cell mediated immunity | | 0.03168 | 2 | BTK/FCER1G | |
| BP | GO:0002889 | regulation of immunoglobulin mediated immune response | | 0.03168 | 2 | BTK/FCER1G | |
| BP | GO:0043030 | regulation of macrophage activation | | 0.03168 | 2 | ITGAM/ITGB2 | |
| BP | GO:0031529 | ruffle organization | | 0.03262 | 2 | PLEK/CSF1R | |
| BP | GO:0032722 | positive regulation of chemokine production | | 0.03262 | 2 | CSF1R/HMOX1 | |
| BP | GO:0051260 | protein homooligomerization | | 0.0333 | 4 | TDO2/ALOX5AP/FCER1G/HMOX1 | |
| BP | GO:0006584 | catecholamine metabolic process | | 0.0333 | 2 | ITGAM/ITGB2 | |
| BP | GO:0009712 | catechol-containing compound metabolic process | | 0.0333 | 2 | ITGAM/ITGB2 | |
| BP | GO:0045123 | cellular extravasation | | 0.0333 | 2 | SELPLG/ITGB2 | |
| BP | GO:0022408 | negative regulation of cell-cell adhesion | | 0.03409 | 3 | IL2RA/CD300A/LILRB1 | |
| BP | GO:0051251 | positive regulation of lymphocyte activation | | 0.03462 | 4 | IL2RA/BTK/CD4/LILRB1 | |
| BP | GO:0031663 | lipopolysaccharide-mediated signaling pathway | | 0.03506 | 2 | CD180/HCK | |
| BP | GO:0035306 | positive regulation of dephosphorylation | | 0.03506 | 2 | PLEK/CD300A | |
| BP | GO:0042130 | negative regulation of T cell proliferation | | 0.03506 | 2 | IL2RA/LILRB1 | |
| BP | GO:0002285 | lymphocyte activation involved in immune response | | 0.03566 | 3 | CD180/FCER1G/LILRB1 | |
| BP | GO:0042267 | natural killer cell mediated cytotoxicity | | 0.03724 | 2 | SERPINB4/LILRB1 | |
| BP | GO:0030098 | lymphocyte differentiation | | 0.03786 | 4 | IL2RA/BTK/FCER1G/CD4 | |
| BP | GO:0030888 | regulation of B cell proliferation | | 0.03806 | 2 | BTK/CD300A | |
| BP | GO:0046173 | polyol biosynthetic process | | 0.03806 | 2 | PLEK/HRH1 | |
| BP | GO:0050864 | regulation of B cell activation | | 0.04002 | 3 | BTK/SAMSN1/CD300A | |
| BP | GO:1903708 | positive regulation of hemopoiesis | | 0.04002 | 3 | IL2RA/BTK/CD4 | |
| BP | GO:0006968 | cellular defense response | | 0.04015 | 2 | NCF2/TYROBP | |
| BP | GO:0043112 | receptor metabolic process | | 0.04017 | 3 | ITGB2/FCER1G/LILRB1 | |
| BP | GO:0071248 | cellular response to metal ion | | 0.04017 | 3 | ALOX5AP/HMOX1/CYBB | |
| BP | GO:0002228 | natural killer cell mediated immunity | | 0.04083 | 2 | SERPINB4/LILRB1 | |
| BP | GO:0032623 | interleukin-2 production | | 0.04083 | 2 | FCER1G/CD4 | |
| BP | GO:0002532 | production of molecular mediator involved in inflammatory response | | 0.0418 | 2 | BTK/FCER1G | |
| BP | GO:0046686 | response to cadmium ion | | 0.0418 | 2 | HMOX1/CYBB | |
| BP | GO:0045670 | regulation of osteoclast differentiation | | 0.04293 | 2 | TYROBP/LILRB1 | |
| BP | GO:0034121 | regulation of toll-like receptor signaling pathway | | 0.04407 | 2 | CD300 LF/CD300A | |
| BP | GO:0002709 | regulation of T cell mediated immunity | | 0.04491 | 2 | CLC/LILRB1 | |
| BP | GO:0032387 | negative regulation of intracellular transport | | 0.04491 | 2 | CD300A/HMOX1 | |
| BP | GO:0072376 | protein activation cascade | | 0.04491 | 3 | F13A1/CFI/C1QB | |
| BP | GO:1903531 | negative regulation of secretion by cell | | 0.04578 | 3 | CD300A/HMOX1/LILRB1 | |
| BP | GO:0061515 | myeloid cell development | | 0.04578 | 2 | TYROBP/LILRB1 | |
| BP | GO:0043900 | regulation of multiorganism process | | 0.04578 | 4 | CD180/IL2RA/CD4/LILRB1 | |
| BP | GO:1903532 | positive regulation of secretion by cell | | 0.04646 | 4 | ITGAM/ITGB2/FCER1G/CSF1R | |
| BP | GO:0050688 | regulation of defense response to virus | | 0.04673 | 2 | IL2RA/LILRB1 | |
| BP | GO:0031348 | negative regulation of defense response | | 0.04678 | 3 | SERPINB4/IL2RA/LILRB1 | |
| BP | GO:0071222 | cellular response to lipopolysaccharide | | 0.04678 | 3 | CD180/HCK/LILRB1 | |
| BP | GO:0097164 | ammonium ion metabolic process | | 0.04678 | 3 | ITGAM/PLA2G7/ITGB2 | |
| BP | GO:0045766 | positive regulation of angiogenesis | | 0.04723 | 3 | ITGB2/HMOX1/CYBB | |
| BP | GO:0043647 | inositol phosphate metabolic process | | 0.04723 | 2 | PLEK/HRH1 | |
| BP | GO:0050870 | positive regulation of T cell activation | | 0.04821 | 3 | IL2RA/CD4/LILRB1 | |
| CC | GO:0070821 | tertiary granule membrane | | 5.80E-05 | 5 | ITGAM/ITGB2/FCER1G/CD300A/CYBB | |
| CC | GO:0009897 | external side of plasma membrane | | 5.80E-05 | 8 | ITGAM/IL2RA/MS4A2/FOLR2/ITGB2/FCER1G/CD4/LILRB1 | |
| CC | GO:0070820 | tertiary granule | | 5.80E-05 | 6 | FPR1/ITGAM/ITGB2/FCER1G/CD300A/CYBB | |
| CC | GO:0044853 | plasma membrane raft | | 7.19E-05 | 5 | ITGAM/HCK/SELPLG/ITGB2/HMOX1 | |
| CC | GO:0030667 | secretory granule membrane | | 7.19E-05 | 7 | FPR1/ITGAM/ITGB2/FCER1G/TYROBP/CD300A/CYBB | |
| CC | GO:0045121 | membrane raft | | 7.19E-05 | 7 | ITGAM/HCK/BTK/SELPLG/ITGB2/CD4/HMOX1 | |
| CC | GO:0098857 | membrane microdomain | | 7.19E-05 | 7 | ITGAM/HCK/BTK/SELPLG/ITGB2/CD4/HMOX1 | |
| CC | GO:0098589 | membrane region | | 7.93E-05 | 7 | ITGAM/HCK/BTK/SELPLG/ITGB2/CD4/HMOX1 | |
| CC | GO:0101003 | ficolin-1-rich granule membrane | | 0.00011 | 4 | FPR1/ITGB2/FCER1G/CD300A | |
| CC | GO:0101002 | ficolin-1-rich granule | | 0.00057 | 5 | FPR1/HK3/ITGB2/FCER1G/CD300A | |
| CC | GO:0043020 | NADPH oxidase complex | | 0.00276 | 2 | NCF2/CYBB | |
| CC | GO:0031012 | extracellular matrix | | 0.0063 | 6 | CLC/F13A1/CD180/COL10A1/C1QB/CPA3 | |
| CC | GO:0062023 | collagen-containing extracellular matrix | | 0.00655 | 5 | CLC/F13A1/COL10A1/C1QB/CPA3 | |
| CC | GO:0035579 | specific granule membrane | | 0.00769 | 3 | ITGAM/ITGB2/CYBB | |
| CC | GO:0008305 | integrin complex | | 0.01476 | 2 | ITGAM/ITGB2 | |
| CC | GO:0098636 | protein complex involved in cell adhesion | | 0.01652 | 2 | ITGAM/ITGB2 | |
| CC | GO:0042581 | specific granule | | 0.03129 | 3 | ITGAM/ITGB2/CYBB | |
| CC | GO:0098802 | plasma membrane receptor complex | | 0.04337 | 3 | ITGAM/ITGB2/CD4 | |
| MF | GO:0019865 | immunoglobulin binding | | 0.0036 | 3 | MS4A2/FCER1G/CD4 | |
| MF | GO:0048020 | CCR chemokine receptor binding | | 0.01194 | 3 | CCL18/CCL13/CCL8 | |
| MF | GO:0008009 | chemokine activity | | 0.01194 | 3 | CCL18/CCL13/CCL8 | |
| MF | GO:0016175 | superoxide-generating NADPH oxidase activity | | 0.01255 | 2 | NCF2/CYBB | |
| MF | GO:0001846 | opsonin binding | | 0.01442 | 2 | ITGAM/ITGB2 | |
| MF | GO:0042379 | chemokine receptor binding | | 0.01442 | 3 | CCL18/CCL13/CCL8 | |
| MF | GO:0050664 | oxidoreductase activity, acting on NAD(P)H, oxygen as acceptor | | 0.01553 | 2 | NCF2/CYBB | |
| MF | GO:0001848 | complement binding | | 0.01872 | 2 | ITGAM/ITGB2 | |
| MF | GO:0031406 | carboxylic acid binding | | 0.01872 | 4 | TDO2/ALOX5AP/LYVE1/FOLR2 | |
| MF | GO:0072341 | modified amino acid binding | | 0.01872 | 3 | FOLR2/CD300 LF/CD300A | |
| MF | GO:0043177 | organic acid binding | | 0.02096 | 4 | TDO2/ALOX5AP/LYVE1/FOLR2 | |
| MF | GO:0005543 | phospholipid binding | | 0.03954 | 5 | PLA2G7/BTK/PLEK/CD300 LF/CD300A | |
| MF | GO:0019955 | cytokine binding | | 0.03954 | 3 | IL2RA/CD4/CSF1R | |
| MF | GO:0042287 | MHC protein binding | | 0.03954 | 2 | CD4/LILRB1 | |
| MF | GO:0020037 | heme binding | | 0.04008 | 3 | TDO2/HMOX1/CYBB | |
| MF | GO:0001664 | G protein-coupled receptor binding | | 0.04008 | 4 | FPR1/CCL18/CCL13/CCL8 | |
| MF | GO:0001784 | phosphotyrosine residue binding | | 0.04008 | 2 | HCK/SAMSN1 | |
| MF | GO:0005126 | cytokine receptor binding | | 0.04021 | 4 | CCL18/CCL13/CD300 LF/CCL8 | |
| MF | GO:0046906 | tetrapyrrole binding | | 0.04021 | 3 | TDO2/HMOX1/CYBB | |
| MF | GO:0004715 | nonmembrane spanning protein tyrosine kinase activity | | 0.0448 | 2 | HCK/BTK | |
| MF | GO:0045309 | protein phosphorylated amino acid binding | | 0.04826 | 2 | HCK/SAMSN1 | |
| BP, biological process | | |  |  |  |  |  |
| CC, cellular component | | |  |  |  |  |  |
| MF, molecular function  adj. p-value, adjusted p-value | | |  |  |  |  |  |

| **Table S3. GO functional annotation enrichment analysis of downregulated co-DEGs in CRSwNP** | | | | | | |
| --- | --- | --- | --- | --- | --- | --- |
| **Ontology** | **ID** | **Description** | | **adj. p-value** | **Count** | **Gene ID** |
| BP | GO:0001580 | detection of chemical stimulus involved in sensory perception of bitter taste | | 0.0126483 | 3 | LPO/PIP/AZGP1 |
| BP | GO:0050913 | sensory perception of bitter taste | | 0.0126483 | 3 | LPO/PIP/AZGP1 |
| BP | GO:0050912 | detection of chemical stimulus involved in sensory perception of taste | | 0.0126483 | 3 | LPO/PIP/AZGP1 |
| BP | GO:0050909 | sensory perception of taste | | 0.0274413 | 3 | LPO/PIP/AZGP1 |
| BP | GO:0015893 | drug transport | | 0.0329499 | 4 | SLC22A3/FOLR1/CA2/TCN1 |
| BP | GO:0060749 | mammary gland alveolus development | | 0.0329499 | 2 | EGF/ERBB4 |
| BP | GO:0061377 | mammary gland lobule development | | 0.0329499 | 2 | EGF/ERBB4 |
| BP | GO:0001894 | tissue homeostasis | | 0.0329499 | 4 | PIP/AZGP1/CA2/TFF1 |
| BP | GO:0022600 | digestive system process | | 0.0363697 | 3 | SLC5A1/KCNN4/TFF1 |
| BP | GO:0015711 | organic anion transport | | 0.0477567 | 5 | FABP4/FOLR1/CA2/KCNN4/SLCO1A2 |
| BP | GO:0048871 | multicellular organismal homeostasis | | 0.0483033 | 5 | FABP4/PIP/AZGP1/CA2/TFF1 |
| CC | GO:0016323 | basolateral plasma membrane | | 0.0281485 | 4 | LPO/ERBB4/FOLR1/CA2 |
| MF | GO:0015378 | sodium: chloride symporter activity | | 0.0094745 | 2 | SLC22A3/SLC12A2 |
| MF | GO:0015373 | anion: sodium symporter activity | | 0.0094745 | 2 | SLC22A3/SLC12A2 |
| MF | GO:0015370 | solute: sodium symporter activity | | 0.0094745 | 3 | SLC22A3/SLC12A2/SLC5A1 |
| MF | GO:0015377 | cation: chloride symporter activity | | 0.0097469 | 2 | SLC22A3/SLC12A2 |
| MF | GO:0015291 | secondary active transmembrane transporter activity | | 0.0097469 | 4 | SLC22A3/SLC12A2/SLC5A1/SLCO1A2 |
| MF | GO:0015296 | anion: cation symporter activity | | 0.0097469 | 2 | SLC22A3/SLC12A2 |
| MF | GO:0015294 | solute: cation symporter activity | | 0.0107746 | 3 | SLC22A3/SLC12A2/SLC5A1 |
| MF | GO:0046873 | metal ion transmembrane transporter activity | | 0.0107746 | 5 | SLC22A3/SLC12A2/SLC5A1/SLC31A2/KCNN4 |
| MF | GO:0008519 | ammonium transmembrane transporter activity | | 0.0178109 | 2 | SLC22A3/SLC12A2 |
| MF | GO:0005154 | epidermal growth factor receptor binding | | 0.0178109 | 2 | EGF/ERBB4 |
| MF | GO:0015293 | symporter activity | | 0.0187919 | 3 | SLC22A3/SLC12A2/SLC5A1 |
| MF | GO:0015081 | sodium ion transmembrane transporter activity | | 0.020156 | 3 | SLC22A3/SLC12A2/SLC5A1 |
| MF | GO:0022804 | active transmembrane transporter activity | | 0.0225491 | 4 | SLC22A3/SLC12A2/SLC5A1/SLCO1A2 |
| MF | GO:1901618 | organic hydroxy compound transmembrane transporter activity | | 0.0225491 | 2 | SLC22A3/SLCO1A2 |
| MF | GO:0015077 | monovalent inorganic cation transmembrane transporter activity | | 0.0293933 | 4 | SLC22A3/SLC12A2/SLC5A1/KCNN4 |
| MF | GO:0046934 | phosphatidylinositol-4,5-bisphosphate 3-kinase activity | | 0.046408 | 2 | EGF/ERBB4 |
| MF | GO:0052813 | phosphatidylinositol bisphosphate kinase activity | | 0.0475075 | 2 | EGF/ERBB4 |
| BP, biological process | | |  |  |  |  |
| CC, cellular component | | |  |  |  |  |
| MF, molecular function  adj. p-value, adjusted p-value | | |  |  |  |  |

| **Table S4. KEGG cell signaling pathway analysis of co-DEGs in CRSwNP** | | | | |
| --- | --- | --- | --- | --- |
| **ID** | **Description** | **adj. p-value** | **Count** | **Gene ID** |
| hsa05150 | *Staphylococcus aureus* infection | 0.0001847 | 6 | FPR1/ITGAM/CFI/C1QB/SELPLG/ITGB2 |
| hsa04610 | Complement and coagulation cascades | 0.000961 | 5 | F13A1/ITGAM/CFI/C1QB/ITGB2 |
| hsa04061 | Viral protein interaction with cytokine and cytokine receptor | 0.0014074 | 5 | CCL18/CCL13/IL2RA/CCL8/CSF1R |
| hsa04380 | Osteoclast differentiation | 0.0027672 | 5 | NCF2/BTK/TYROBP/CSF1R/LILRB1 |
| hsa04664 | Fc epsilon RI signaling pathway | 0.0027672 | 4 | ALOX5AP/BTK/MS4A2/FCER1G |
| hsa05140 | Leishmaniasis | 0.0037333 | 4 | NCF2/ITGAM/ITGB2/CYBB |
| hsa04640 | Hematopoietic cell lineage | 0.0083523 | 4 | ITGAM/IL2RA/CD4/CSF1R |
| hsa04670 | Leukocyte transendothelial migration | 0.0105857 | 4 | NCF2/ITGAM/ITGB2/CYBB |
| hsa04060 | Cytokine-cytokine receptor interaction | 0.0105857 | 6 | CCL18/CCL13/IL2RA/CCL8/CD4/CSF1R |
| hsa05221 | Acute myeloid leukemia | 0.0218756 | 3 | ITGAM/BCL2A1/CSF1R |
| hsa04514 | Cell adhesion molecules (CAMs) | 0.0229481 | 4 | ITGAM/SELPLG/ITGB2/CD4 |
| hsa04145 | Phagosome | 0.0237347 | 4 | NCF2/ITGAM/ITGB2/CYBB |
| hsa05133 | Pertussis | 0.0240809 | 3 | ITGAM/C1QB/ITGB2 |
| hsa05310 | Asthma | 0.0411127 | 2 | MS4A2/FCER1G |
| hsa04062 | Chemokine signaling pathway | 0.0411127 | 4 | CCL18/CCL13/HCK/CCL8 |
| hsa05146 | Amoebiasis | 0.0441197 | 3 | SERPINB4/ITGAM/ITGB2 |
| hsa04064 | NF-kappa B signaling pathway | 0.0441197 | 3 | CCL13/BCL2A1/BTK |
| hsa04066 | HIF-1 signaling pathway | 0.0452703 | 3 | HK3/HMOX1/CYBB |
| hsa04015 | Rap1 signaling pathway | 0.0452703 | 4 | FPR1/ITGAM/ITGB2/CSF1R |
| hsa05340 | Primary immunodeficiency | 0.0452703 | 2 | BTK/CD4 |
| hsa04216 | Ferroptosis | 0.0475927 | 2 | HMOX1/CYBB |
| upregulated co-DEGs | |  |  |  |
|  |  |  |  |  |
| **ID** | **Description** | **adj. p-value** | **Count** | **Gene ID** |
| hsa04976 | Bile secretion | 0.0199821 | 3 | CA2/SLCO1A2SLC5A1 |
| hsa04970 | Salivary secretion | 0.0199821 | 3 | LPO/KCNN4SLC12A2 |
| downregulated co-DEGs | |  |  |  |

**Table S5. GO functional annotation enrichment analysis of the hub genes in CRSwNP**

| ONTO  LOGY | | ID | Description | | Gene Ratio | | BgRatio | p-value | adj. p-value | q value | geneID | Count |
| --- | --- | --- | --- | --- | --- | --- | --- | --- | --- | --- | --- | --- |
| BP | | GO:0042554 | superoxide anion generation | | | 3/12 | 31/18493 | 9.29E-07 | 0.0003853 | 0.0001945 | NCF2/ITGB2/CYBB | 3 |
| BP | | GO:0045730 | respiratory burst | | | 3/12 | 33/18493 | 1.13E-06 | 0.0003853 | 0.0001945 | NCF2/HCK/CYBB | 3 |
| BP | | GO:0018108 | peptidyl-tyrosine phosphorylation | | | 5/12 | 387/18493 | 2.74E-06 | 0.0004874 | 0.000246 | ITGB2/HCK/CSF1R/BTK/CD300A | 5 |
| BP | | GO:0018212 | peptidyl-tyrosine modification | | | 5/12 | 390/18493 | 2.85E-06 | 0.0004874 | 0.000246 | ITGB2/HCK/CSF1R/BTK/CD300A | 5 |
| BP | | GO:0002886 | regulation of myeloid leukocyte mediated immunity | | | 3/12 | 51/18493 | 4.27E-06 | 0.0005844 | 0.000295 | ITGB2/BTK/CD300A | 3 |
| BP | | GO:0043312 | neutrophil degranulation | | | 5/2 | 485/18493 | 8.27E-06 | 0.0006983 | 0.0003525 | ITGB2/FPR1/CD300A/CYBB/HK3 | 5 |
| BP | | GO:0002283 | neutrophil activation involved in immune response | | | 5/12 | 488/18493 | 8.52E-06 | 0.0006983 | 0.0003525 | ITGB2/FPR1/CD300A/CYBB/HK3 | 5 |
| BP | | GO:0042119 | neutrophil activation | | | 5/12 | 498/18493 | 9.40E-06 | 0.0006983 | 0.0003525 | ITGB2/FPR1/CD300A/CYBB/HK3 | 5 |
| BP | | GO:0002446 | neutrophil mediated immunity | | | 5/12 | 499/18493 | 9.49E-06 | 0.0006983 | 0.0003525 | ITGB2/FPR1/CD300A/CYBB/HK3 | 5 |
| BP | | GO:0006801 | superoxide metabolic process | | | 3/12 | 69/18493 | 1.07E-05 | 0.0006983 | 0.0003525 | NCF2/ITGB2/CYBB | 3 |
| BP | | GO:0002758 | innate immune response-activating signal transduction | | | 4/12 | 233/18493 | 1.12E-05 | 0.0006983 | 0.0003525 | ITGB2/HCK/BTK/CD300A | 4 |
| BP | | GO:0002218 | activation of innate immune response | | | 4/12 | 252/18493 | 1.53E-05 | 0.0008717 | 0.00044 | ITGB2/HCK/BTK/CD300A | 4 |
| BP | | GO:1902563 | regulation of neutrophil activation | | | 2/12 | 11/18493 | 2.12E-05 | 0.0011134 | 0.000562 | ITGB2/CD300A | 2 |
| BP | | GO:0031532 | actin cytoskeleton reorganization | | | 3/12 | 96/18493 | 2.88E-05 | 0.0014085 | 0.000711 | HCK/CSF1R/PLEK | 3 |
| BP | | GO:0007229 | integrin-mediated signaling pathway | | | 3/12 | 99/18493 | 3.16E-05 | 0.0014416 | 0.0007277 | ITGB2/HCK/PLEK | 3 |
| BP | | GO:0045089 | positive regulation of innate immune response | | | 4/12 | 309/18493 | 3.40E-05 | 0.0014553 | 0.0007346 | ITGB2/HCK/BTK/CD300A | 4 |
| BP | | GO:0030889 | negative regulation of B cell proliferation | | | 2/12 | 15/18493 | 4.03E-05 | 0.0016231 | 0.0008193 | BTK/CD300A | 2 |
| BP | | GO:0006909 | phagocytosis | | | 4/12 | 342/18493 | 5.06E-05 | 0.0019225 | 0.0009704 | NCF2/ITGB2/HCK/CD300A | 4 |
| BP | | GO:0045088 | regulation of innate immune response | | | 4/12 | 365/18493 | 6.52E-05 | 0.0023467 | 0.0011845 | ITGB2/HCK/BTK/CD300A | 4 |
| BP | | GO:0002224 | toll-like receptor signaling pathway | | | 3/12 | 131/18493 | 7.29E-05 | 0.0024945 | 0.0012591 | ITGB2/BTK/CD300A | 3 |
| BP | | GO:0002429 | immune response-activating cell surface receptor signaling pathway | | | 4/12 | 414/18493 | 0.000106 | 0.0034583 | 0.0017456 | FPR1/HCK/BTK/CD300A | 4 |
| BP | | GO:0008360 | regulation of cell shape | | | 3/12 | 151/18493 | 0.000111 | 0.0034583 | 0.0017456 | ITGB2/HCK/CSF1R | 3 |
| BP | | GO:0031349 | positive regulation of defense response | | | 4/12 | 427/18493 | 0.00012 | 0.0035614 | 0.0017977 | ITGB2/HCK/BTK/CD300A | 4 |
| BP | | GO:0002768 | immune response-regulating cell surface receptor signaling pathway | | | 4/12 | 445/18493 | 0.00014 | 0.0039547 | 0.0019962 | FPR1/HCK/BTK/CD300A | 4 |
| BP | | GO:0002888 | positive regulation of myeloid leukocyte mediated immunity | | | 2/12 | 28/18493 | 0.000145 | 0.0039547 | 0.0019962 | ITGB2/BTK | 2 |
| BP | | GO:0050869 | negative regulation of B cell activation | | | 2/12 | 29/18493 | 0.000155 | 0.0040828 | 0.0020609 | BTK/CD300A | 2 |
| BP | | GO:0002221 | pattern recognition receptor signaling pathway | | | 3/12 | 179/18493 | 0.000184 | 0.0046607 | 0.0023526 | ITGB2/BTK/CD300A | 3 |
| BP | | GO:0010922 | positive regulation of phosphatase activity | | | 2/12 | 34/18493 | 0.000214 | 0.0050489 | 0.0025485 | PLEK/CD300A | 2 |
| BP | | GO:2000249 | regulation of actin cytoskeleton reorganization | | | 2/12 | 34/18493 | 0.000214 | 0.0050489 | 0.0025485 | HCK/CSF1R | 2 |
| BP | | GO:0002755 | MyD88-dependent toll-like receptor signaling pathway | | | 2/12 | 36/18493 | 0.00024 | 0.0053039 | 0.0026773 | BTK/CD300A | 2 |
| BP | | GO:0002703 | regulation of leukocyte mediated immunity | | | 3/12 | 196/18493 | 0.00024 | 0.0053039 | 0.0026773 | ITGB2/BTK/CD300A | 3 |
| BP | | GO:0038083 | peptidyl-tyrosine autophosphorylation | | | 2/12 | 37/18493 | 0.000254 | 0.005426 | 0.0027389 | HCK/BTK | 2 |
| BP | | GO:0002699 | positive regulation of immune effector process | | | 3/12 | 209/18493 | 0.00029 | 0.0060182 | 0.0030378 | ITGB2/BTK/CD300A | 3 |
| BP | | GO:0043300 | regulation of leukocyte degranulation | | | 2/12 | 42/18493 | 0.000328 | 0.0065902 | 0.0033265 | ITGB2/CD300A | 2 |
| BP | | GO:0043303 | mast cell degranulation | | | 2/12 | 44/18493 | 0.00036 | 0.0069557 | 0.003511 | BTK/CD300A | 2 |
| BP | | GO:0046777 | protein autophosphorylation | | | 3/12 | 227/18493 | 0.00037 | 0.0069557 | 0.003511 | HCK/CSF1R/BTK | 3 |
| BP | | GO:0002279 | mast cell activation involved in immune response | | | 2/12 | 45/18493 | 0.000376 | 0.0069557 | 0.003511 | BTK/CD300A | 2 |
| BP | | GO:0002448 | mast cell mediated immunity | | | 2/12 | 46/18493 | 0.000393 | 0.0070779 | 0.0035727 | BTK/CD300A | 2 |
| BP | | GO:0050730 | regulation of peptidyl-tyrosine phosphorylation | | | 3/12 | 245/18493 | 0.000463 | 0.0081127 | 0.004095 | ITGB2/CSF1R/CD300A | 3 |
| BP | | GO:0031529 | ruffle organization | | | 2/12 | 53/18493 | 0.000522 | 0.0089298 | 0.0045075 | CSF1R/PLEK | 2 |
| BP | | GO:0035306 | positive regulation of dephosphorylation | | | 2/12 | 56/18493 | 0.000583 | 0.0097257 | 0.0049092 | PLEK/CD300A | 2 |
| BP | | GO:0045576 | mast cell activation | | | 2/12 | 57/18493 | 0.000604 | 0.0098358 | 0.0049648 | BTK/CD300A | 2 |
| BP | | GO:0030888 | regulation of B cell proliferation | | | 2/12 | 59/18493 | 0.000647 | 0.0101781 | 0.0051376 | BTK/CD300A | 2 |
| BP | | GO:0072593 | reactive oxygen species metabolic process | | | 3/12 | 276/18493 | 0.000655 | 0.0101781 | 0.0051376 | NCF2/ITGB2/CYBB | 3 |
| BP | | GO:0032418 | lysosome localization | | | 2/12 | 65/18493 | 0.000785 | 0.0119296 | 0.0060217 | BTK/CD300A | 2 |
| BP | | GO:0032945 | negative regulation of mononuclear cell proliferation | | | 2/12 | 70/18493 | 0.00091 | 0.0132376 | 0.006682 | BTK/CD300A | 2 |
| BP | | GO:0050672 | negative regulation of lymphocyte proliferation | | | 2/12 | 70/18493 | 0.00091 | 0.0132376 | 0.006682 | BTK/CD300A | 2 |
| BP | | GO:0070664 | negative regulation of leukocyte proliferation | | | 2/12 | 75/18493 | 0.001043 | 0.0148672 | 0.0075045 | BTK/CD300A | 2 |
| BP | | GO:0032956 | regulation of actin cytoskeleton organization | | | 3/12 | 331/18493 | 0.001109 | 0.0154774 | 0.0078126 | HCK/CSF1R/PLEK | 3 |
| BP | | GO:0050764 | regulation of phagocytosis | | | 2/12 | 80/18493 | 0.001186 | 0.0162234 | 0.0081891 | HCK/CD300A | 2 |
| BP | | GO:0140029 | exocytic process | | | 2/12 | 82/18493 | 0.001245 | 0.0167036 | 0.0084315 | PLEK/BTK | 2 |
| BP | | GO:0046427 | positive regulation of JAK-STAT cascade | | | 2/12 | 85/18493 | 0.001337 | 0.0175917 | 0.0088798 | CSF1R/CD300A | 2 |
| BP | | GO:1904894 | positive regulation of STAT cascade | | | 2/12 | 88/18493 | 0.001432 | 0.0184871 | 0.0093318 | CSF1R/CD300A | 2 |
| BP | | GO:0042100 | B cell proliferation | | | 2/12 | 91/18493 | 0.001531 | 0.0190368 | 0.0096092 | BTK/CD300A | 2 |
| BP | | GO:0048010 | vascular endothelial growth factor receptor signaling pathway | | | 2/12 | 91/18493 | 0.001531 | 0.0190368 | 0.0096092 | NCF2/CYBB | 2 |
| BP | | GO:0032970 | regulation of actin filament-based process | | | 3/12 | 376/18493 | 0.0016 | 0.0195469 | 0.0098667 | HCK/CSF1R/PLEK | 3 |
| BP | | GO:0050867 | positive regulation of cell activation | | | 3/12 | 384/18493 | 0.0017 | 0.0203994 | 0.010297 | ITGB2/PLEK/BTK | 3 |
| BP | | GO:0051090 | regulation of DNA-binding transcription factor activity | | | 3/12 | 412/18493 | 0.002079 | 0.0245201 | 0.012377 | ITGB2/HCK/BTK | 3 |
| BP | | GO:0050727 | regulation of inflammatory response | | | 3/12 | 425/18493 | 0.002272 | 0.0262891 | 0.01327 | ITGB2/HCK/BTK | 3 |
| BP | | GO:0006911 | phagocytosis, engulfment | | | 2/12 | 112/18493 | 0.002306 | 0.0262891 | 0.01327 | ITGB2/CD300A | 2 |
| BP | | GO:1903409 | reactive oxygen species biosynthetic process | | | 2/12 | 115/18493 | 0.002429 | 0.0270515 | 0.0136548 | ITGB2/CYBB | 2 |
| BP | | GO:0002697 | regulation of immune effector process | | | 3/12 | 439/18493 | 0.002492 | 0.0270515 | 0.0136548 | ITGB2/BTK/CD300A | 3 |
| BP | | GO:0019932 | second-messenger-mediated signaling | | | 3/12 | 439/18493 | 0.002492 | 0.0270515 | 0.0136548 | FPR1/PLEK/BTK | 3 |
| BP | | GO:0099024 | plasma membrane invagination | | | 2/12 | 121/18493 | 0.002685 | 0.0286922 | 0.014483 | ITGB2/CD300A | 2 |
| BP | | GO:0050853 | B cell receptor signaling pathway | | | 2/12 | 122/18493 | 0.002728 | 0.0287113 | 0.0144926 | BTK/CD300A | 2 |
| BP | | GO:0006091 | generation of precursor metabolites and energy | | | 3/12 | 471/18493 | 0.003043 | 0.029874 | 0.0150795 | NCF2/CYBB/HK3 | 3 |
| BP | | GO:0010324 | membrane invagination | | | 2/12 | 129/18493 | 0.003044 | 0.029874 | 0.0150795 | ITGB2/CD300A | 2 |
| BP | | GO:0046425 | regulation of JAK-STAT cascade | | | 2/12 | 129/18493 | 0.003044 | 0.029874 | 0.0150795 | CSF1R/CD300A | 2 |
| BP | | GO:0022604 | regulation of cell morphogenesis | | | 3/12 | 474/18493 | 0.003098 | 0.029874 | 0.0150795 | ITGB2/HCK/CSF1R | 3 |
| BP | | GO:0002705 | positive regulation of leukocyte mediated immunity | | | 2/12 | 131/18493 | 0.003137 | 0.029874 | 0.0150795 | ITGB2/BTK | 2 |
| BP | | GO:0007498 | mesoderm development | | | 2/12 | 131/18493 | 0.003137 | 0.029874 | 0.0150795 | HCK/BTK | 2 |
| BP | | GO:0050900 | leukocyte migration | | | 3/12 | 478/18493 | 0.003172 | 0.029874 | 0.0150795 | ITGB2/HCK/CD300A | 3 |
| BP | | GO:0050729 | positive regulation of inflammatory response | | | 2/12 | 133/18493 | 0.003232 | 0.029874 | 0.0150795 | ITGB2/BTK | 2 |
| BP | | GO:1904892 | regulation of STAT cascade | | | 2/12 | 133/18493 | 0.003232 | 0.029874 | 0.0150795 | CSF1R/CD300A | 2 |
| BP | | GO:0097530 | granulocyte migration | | | 2/12 | 134/18493 | 0.00328 | 0.0299115 | 0.0150985 | ITGB2/CD300A | 2 |
| BP | | GO:0051250 | negative regulation of lymphocyte activation | | | 2/12 | 137/18493 | 0.003425 | 0.0308262 | 0.0155602 | BTK/CD300A | 2 |
| BP | | GO:0071900 | regulation of protein serine/threonine kinase activity | | | 3/12 | 496/18493 | 0.003522 | 0.0312356 | 0.0157668 | FPR1/CSF1R/CD300A | 3 |
| BP | | GO:0060627 | regulation of vesicle-mediated transport | | | 3/12 | 498/18493 | 0.003562 | 0.0312356 | 0.0157668 | ITGB2/HCK/CD300A | 3 |
| BP | | GO:0051092 | positive regulation of NF-kappaB transcription factor activity | | | 2/12 | 146/18493 | 0.003879 | 0.0335861 | 0.0169533 | ITGB2/BTK | 2 |
| BP | | GO:0007259 | JAK-STAT cascade | | | 2/12 | 147/18493 | 0.003931 | 0.0336116 | 0.0169661 | CSF1R/CD300A | 2 |
| BP | | GO:1903305 | regulation of regulated secretory pathway | | | 2/12 | 148/18493 | 0.003984 | 0.0336393 | 0.0169801 | ITGB2/CD300A | 2 |
| BP | | GO:0050852 | T cell receptor signaling pathway | | | 2/12 | 150/18493 | 0.004089 | 0.0341117 | 0.0172186 | BTK/CD300A | 2 |
| BP | | GO:0097696 | STAT cascade | | | 2/12 | 151/18493 | 0.004143 | 0.0341407 | 0.0172332 | CSF1R/CD300A | 2 |
| BP | | GO:0002695 | negative regulation of leukocyte activation | | | 2/12 | 164/18493 | 0.004867 | 0.0396279 | 0.020003 | BTK/CD300A | 2 |
| BP | | GO:0010921 | regulation of phosphatase activity | | | 2/12 | 172/18493 | 0.005339 | 0.0429638 | 0.0216869 | PLEK/CD300A | 2 |
| BP | | GO:0008064 | regulation of actin polymerization or depolymerization | | | 2/12 | 178/18493 | 0.005707 | 0.0446112 | 0.0225184 | HCK/PLEK | 2 |
| BP | | GO:0030832 | regulation of actin filament length | | | 2/12 | 179/18493 | 0.005769 | 0.0446112 | 0.0225184 | HCK/PLEK | 2 |
| BP | | GO:0022900 | electron transport chain | | | 2/12 | 182/18493 | 0.005958 | 0.0446112 | 0.0225184 | NCF2/CYBB | 2 |
| BP | | GO:0050864 | regulation of B cell activation | | | 2/12 | 183/18493 | 0.006022 | 0.0446112 | 0.0225184 | BTK/CD300A | 2 |
| BP | | GO:0050866 | negative regulation of cell activation | | | 2/12 | 184/18493 | 0.006086 | 0.0446112 | 0.0225184 | BTK/CD300A | 2 |
| BP | | GO:0071248 | cellular response to metal ion | | | 2/12 | 184/18493 | 0.006086 | 0.0446112 | 0.0225184 | ALOX5AP/CYBB | 2 |
| BP | | GO:0038093 | Fc receptor signaling pathway | | | 2/12 | 187/18493 | 0.00628 | 0.0446112 | 0.0225184 | HCK/BTK | 2 |
| BP | | GO:0002679 | respiratory burst involved in defense response | | | 1/12 | 10/18493 | 0.006472 | 0.0446112 | 0.0225184 | HCK | 1 |
| BP | | GO:0002863 | positive regulation of inflammatory response to antigenic stimulus | | | 1/12 | 10/18493 | 0.006472 | 0.0446112 | 0.0225184 | BTK | 1 |
| BP | | GO:0002887 | negative regulation of myeloid leukocyte mediated immunity | | | 1/12 | 10/18493 | 0.006472 | 0.0446112 | 0.0225184 | CD300A | 1 |
| BP | | GO:0051133 | regulation of NK T cell activation | | | 1/12 | 10/18493 | 0.006472 | 0.0446112 | 0.0225184 | CD300A | 1 |
| BP | | GO:0097529 | myeloid leukocyte migration | | | 2/12 | 199/18493 | 0.007083 | 0.0446112 | 0.0225184 | ITGB2/CD300A | 2 |
| BP | | GO:0001821 | histamine secretion | | | 1/12 | 11/18493 | 0.007117 | 0.0446112 | 0.0225184 | BTK | 1 |
| BP | | GO:0002523 | leukocyte migration involved in inflammatory response | | | 1/12 | 11/18493 | 0.007117 | 0.0446112 | 0.0225184 | ITGB2 | 1 |
| BP | | GO:0042535 | positive regulation of tumor necrosis factor biosynthetic process | | | 1/12 | 11/18493 | 0.007117 | 0.0446112 | 0.0225184 | CYBB | 1 |
| BP | | GO:0043301 | negative regulation of leukocyte degranulation | | | 1/12 | 11/18493 | 0.007117 | 0.0446112 | 0.0225184 | CD300A | 1 |
| BP | | GO:0045217 | cell-cell junction maintenance | | | 1/12 | 11/18493 | 0.007117 | 0.0446112 | 0.0225184 | CSF1R | 1 |
| BP | | GO:0045766 | positive regulation of angiogenesis | | | 2/12 | 200/18493 | 0.007152 | 0.0446112 | 0.0225184 | ITGB2/CYBB | 2 |
| BP | | GO:1902905 | positive regulation of supramolecular fiber organization | | | 2/12 | 200/18493 | 0.007152 | 0.0446112 | 0.0225184 | HCK/PLEK | 2 |
| BP | | GO:0017157 | regulation of exocytosis | | | 2/12 | 204/18493 | 0.007431 | 0.0446112 | 0.0225184 | ITGB2/CD300A | 2 |
| BP | | GO:0050670 | regulation of lymphocyte proliferation | | | 2/12 | 205/18493 | 0.007502 | 0.0446112 | 0.0225184 | BTK/CD300A | 2 |
| BP | | GO:0008154 | actin polymerization or depolymerization | | | 2/12 | 206/18493 | 0.007573 | 0.0446112 | 0.0225184 | HCK/PLEK | 2 |
| BP | | GO:0032944 | regulation of mononuclear cell proliferation | | | 2/12 | 206/18493 | 0.007573 | 0.0446112 | 0.0225184 | BTK/CD300A | 2 |
| BP | | GO:0035303 | regulation of dephosphorylation | | | 2/12 | 206/18493 | 0.007573 | 0.0446112 | 0.0225184 | PLEK/CD300A | 2 |
| BP | | GO:0071241 | cellular response to inorganic substance | | | 2/12 | 207/18493 | 0.007644 | 0.0446112 | 0.0225184 | ALOX5AP/CYBB | 2 |
| BP | | GO:0002864 | regulation of acute inflammatory response to antigenic stimulus | | | 1/12 | 12/18493 | 0.007761 | 0.0446112 | 0.0225184 | BTK | 1 |
| BP | | GO:0033004 | negative regulation of mast cell activation | | | 1/12 | 12/18493 | 0.007761 | 0.0446112 | 0.0225184 | CD300A | 1 |
| BP | | GO:0033623 | regulation of integrin activation | | | 1/12 | 12/18493 | 0.007761 | 0.0446112 | 0.0225184 | PLEK | 1 |
| BP | | GO:0036005 | response to macrophage colony-stimulating factor | | | 1/12 | 12/18493 | 0.007761 | 0.0446112 | 0.0225184 | CSF1R | 1 |
| BP | | GO:0036006 | cellular response to macrophage colony-stimulating factor stimulus | | | 1/12 | 12/18493 | 0.007761 | 0.0446112 | 0.0225184 | CSF1R | 1 |
| BP | | GO:0045579 | positive regulation of B cell differentiation | | | 1/12 | 12/18493 | 0.007761 | 0.0446112 | 0.0225184 | BTK | 1 |
| BP | | GO:0051132 | NK T cell activation | | | 1/12 | 12/18493 | 0.007761 | 0.0446112 | 0.0225184 | CD300A | 1 |
| BP | | GO:0051608 | histamine transport | | | 1/12 | 12/18493 | 0.007761 | 0.0446112 | 0.0225184 | BTK | 1 |
| BP | | GO:0070493 | thrombin-activated receptor signaling pathway | | | 1/12 | 12/18493 | 0.007761 | 0.0446112 | 0.0225184 | PLEK | 1 |
| BP | | GO:0070663 | regulation of leukocyte proliferation | | | 2/12 | 216/18493 | 0.008298 | 0.0463662 | 0.0234043 | BTK/CD300A | 2 |
| BP | | GO:0002430 | complement receptor mediated signaling pathway | | | 1/12 | 13/18493 | 0.008406 | 0.0463662 | 0.0234043 | FPR1 | 1 |
| BP | | GO:0030836 | positive regulation of actin filament depolymerization | | | 1/12 | 13/18493 | 0.008406 | 0.0463662 | 0.0234043 | PLEK | 1 |
| BP | | GO:0060099 | regulation of phagocytosis, engulfment | | | 1/12 | 13/18493 | 0.008406 | 0.0463662 | 0.0234043 | CD300A | 1 |
| BP | | GO:0090197 | positive regulation of chemokine secretion | | | 1/12 | 13/18493 | 0.008406 | 0.0463662 | 0.0234043 | CSF1R | 1 |
| BP | | GO:0019722 | calcium-mediated signaling | | | 2/12 | 220/18493 | 0.008596 | 0.0466642 | 0.0235547 | PLEK/BTK | 2 |
| BP | | GO:0051495 | positive regulation of cytoskeleton organization | | | 2/12 | 220/18493 | 0.008596 | 0.0466642 | 0.0235547 | HCK/PLEK | 2 |
| BP | | GO:0034331 | cell junction maintenance | | | 1/12 | 14/18493 | 0.009049 | 0.0476141 | 0.0240342 | CSF1R | 1 |
| BP | | GO:0071361 | cellular response to ethanol | | | 1/12 | 14/18493 | 0.009049 | 0.0476141 | 0.0240342 | CYBB | 1 |
| BP | | GO:0150078 | positive regulation of neuroinflammatory response | | | 1/12 | 14/18493 | 0.009049 | 0.0476141 | 0.0240342 | ITGB2 | 1 |
| BP | | GO:1905153 | regulation of membrane invagination | | | 1/12 | 14/18493 | 0.009049 | 0.0476141 | 0.0240342 | CD300A | 1 |
| BP | | GO:1904018 | positive regulation of vasculature development | | | 2/12 | 228/18493 | 0.009207 | 0.0480757 | 0.0242672 | ITGB2/CYBB | 2 |
| BP | | GO:0071216 | cellular response to biotic stimulus | | | 2/12 | 229/18493 | 0.009285 | 0.0481145 | 0.0242868 | HCK/BTK | 2 |
| BP | | GO:0046488 | phosphatidylinositol metabolic process | | | 2/12 | 233/18493 | 0.009599 | 0.0483666 | 0.0244141 | CSF1R/PLEK | 2 |
| BP | | GO:0042976 | activation of Janus kinase activity | | | 1/12 | 15/18493 | 0.009693 | 0.0483666 | 0.0244141 | CD300A | 1 |
| BP | | GO:0050665 | hydrogen peroxide biosynthetic process | | | 1/12 | 15/18493 | 0.009693 | 0.0483666 | 0.0244141 | CYBB | 1 |
| BP | | GO:0071801 | regulation of podosome assembly | | | 1/12 | 15/18493 | 0.009693 | 0.0483666 | 0.0244141 | HCK | 1 |
| BP | | GO:0090196 | regulation of chemokine secretion | | | 1/12 | 15/18493 | 0.009693 | 0.0483666 | 0.0244141 | CSF1R | 1 |
| BP | | GO:0097237 | cellular response to toxic substance | | | 2/12 | 235/18493 | 0.009758 | 0.0483666 | 0.0244141 | ALOX5AP/CYBB | 2 |
| CC | | GO:0070820 | tertiary granule | | | 4/12 | 164/19659 | 2.19E-06 | 0.0001081 | 6.34E-05 | ITGB2/FPR1/CD300A/CYBB | 4 |
| CC | | GO:0101002 | ficolin-1-rich granule | | | 4/12 | 185/19659 | 3.54E-06 | 0.0001081 | 6.34E-05 | ITGB2/FPR1/CD300A/HK3 | 4 |
| CC | | GO:0101003 | ficolin-1-rich granule membrane | | | 3/12 | 61/19659 | 6.13E-06 | 0.0001246 | 7.31E-05 | ITGB2/FPR1/CD300A | 3 |
| CC | | GO:0070821 | tertiary granule membrane | | | 3/12 | 73/19659 | 1.06E-05 | 0.0001609 | 9.44E-05 | ITGB2/CD300A/CYBB | 3 |
| CC | | GO:0030667 | secretory granule membrane | | | 4/12 | 295/19659 | 2.24E-05 | 0.0002284 | 0.000134 | ITGB2/FPR1/CD300A/CYBB | 4 |
| CC | | GO:0043020 | NADPH oxidase complex | | | 2/12 | 12/19659 | 2.25E-05 | 0.0002284 | 0.000134 | NCF2/CYBB | 2 |
| CC | | GO:0045121 | membrane raft | | | 3/12 | 304/19659 | 0.000726 | 0.0055081 | 0.0032317 | ITGB2/HCK/BTK | 3 |
| CC | | GO:0098857 | membrane microdomain | | | 3/12 | 305/19659 | 0.000733 | 0.0055081 | 0.0032317 | ITGB2/HCK/BTK | 3 |
| CC | | GO:0098589 | membrane region | | | 3/12 | 316/19659 | 0.000813 | 0.0055081 | 0.0032317 | ITGB2/HCK/BTK | 3 |
| CC | | GO:0035579 | specific granule membrane | | | 2/12 | 91/19659 | 0.001357 | 0.0082784 | 0.0048571 | ITGB2/CYBB | 2 |
| CC | | GO:0044853 | plasma membrane raft | | | 2/12 | 102/19659 | 0.001701 | 0.0094313 | 0.0055335 | ITGB2/HCK | 2 |
| CC | | GO:1990204 | oxidoreductase complex | | | 2/12 | 110/19659 | 0.001974 | 0.0100347 | 0.0058875 | NCF2/CYBB | 2 |
| CC | | GO:0045335 | phagocytic vesicle | | | 2/12 | 124/19659 | 0.002499 | 0.0117271 | 0.0068804 | NCF2/CYBB | 2 |
| CC | | GO:0042581 | specific granule | | | 2/12 | 160/19659 | 0.004118 | 0.017943 | 0.0105274 | ITGB2/CYBB | 2 |
| CC | | GO:0005767 | secondary lysosome | | | 1/12 | 14/19659 | 0.008515 | 0.0346264 | 0.0203157 | NCF2 | 1 |
| CC | | GO:0030139 | endocytic vesicle | | | 2/12 | 293/19659 | 0.013239 | 0.0479041 | 0.028106 | NCF2/CYBB | 2 |
| CC | | GO:0042629 | mast cell granule | | | 1/12 | 22/19659 | 0.01335 | 0.0479041 | 0.028106 | BTK | 1 |
| MF | | GO:0016175 | superoxide-generating NADPH oxidase activity | | | 2/12 | 11/17632 | 2.33E-05 | 0.0016525 | 0.000833 | NCF2/CYBB | 2 |
| MF | | GO:0050664 | oxidoreductase activity, acting on NAD(P)H, oxygen as acceptor | | | 2/12 | 16/17632 | 5.07E-05 | 0.0017993 | 0.000907 | NCF2/CYBB | 2 |
| MF | | GO:0004713 | protein tyrosine kinase activity | | | 3/12 | 177/17632 | 0.000205 | 0.0048447 | 0.0024421 | HCK/CSF1R/BTK | 3 |
| MF | | GO:0004715 | non-membrane spanning protein tyrosine kinase activity | | | 2/12 | 46/17632 | 0.000432 | 0.007672 | 0.0038673 | HCK/BTK | 2 |
| MF | | GO:0016651 | oxidoreductase activity, acting on NAD(P)H | | | 2/12 | 107/17632 | 0.002314 | 0.0265899 | 0.0134034 | NCF2/CYBB | 2 |
| MF | | GO:0005543 | phospholipid binding | | | 3/12 | 417/17632 | 0.002465 | 0.0265899 | 0.0134034 | PLEK/BTK/CD300A | 3 |
| MF | | GO:0009055 | electron transfer activity | | | 2/12 | 114/17632 | 0.002622 | 0.0265899 | 0.0134034 | NCF2/CYBB | 2 |
| MF | | GO:1901981 | phosphatidylinositol phosphate binding | | | 2/12 | 154/17632 | 0.004723 | 0.0407599 | 0.0205461 | PLEK/BTK | 2 |
| MF | | GO:0005536 | glucose binding | | | 1/12 | 10/17632 | 0.006787 | 0.0407599 | 0.0205461 | HK3 | 1 |
| MF | | GO:0016176 | superoxide-generating NADPH oxidase activator activity | | | 1/12 | 10/17632 | 0.006787 | 0.0407599 | 0.0205461 | NCF2 | 1 |
| MF | | GO:0051400 | BH domain binding | | | 1/12 | 10/17632 | 0.006787 | 0.0407599 | 0.0205461 | BCL2A1 | 1 |
| MF | | GO:0008429 | phosphatidylethanolamine binding | | | 1/12 | 11/17632 | 0.007463 | 0.0407599 | 0.0205461 | CD300A | 1 |
| MF | | GO:0050786 | RAGE receptor binding | | | 1/12 | 11/17632 | 0.007463 | 0.0407599 | 0.0205461 | FPR1 | 1 |
| MF | | GO:0036041 | long-chain fatty acid binding | | | 1/12 | 13/17632 | 0.008814 | 0.04211 | 0.0212267 | ALOX5AP | 1 |
| MF | | GO:0001846 | opsonin binding | | | 1/12 | 14/17632 | 0.00949 | 0.04211 | 0.0212267 | ITGB2 | 1 |
| MF | | GO:0016846 | carbon-sulfur lyase activity | | | 1/12 | 14/17632 | 0.00949 | 0.04211 | 0.0212267 | ALOX5AP | 1 |
| MF | | GO:0035091 | phosphatidylinositol binding | | | 2/12 | 240/17632 | 0.01113 | 0.0464854 | 0.0234322 | PLEK/BTK | 2 |
| BP, biological process | | |  |  |  |  |  |  |  |  |  |  |
| CC, cellular component | | |  |  |  |  |  |  |  |  |  |  |
| MF, molecular function  adj. p-value, adjusted p-value | | |  |  |  |  |  |  |  |  |  |  |

**Table S6. KEGG cell signaling pathway analysis of the hub genes in CRSwNP**

| ID | Description | GeneRatio | BgRatio | p-value | adj. p-value | q value | geneID | Count |
| --- | --- | --- | --- | --- | --- | --- | --- | --- |
| hsa05140 | Leishmaniasis | 3/10 | 77/8018 | 9.74E-05 | 0.0055499 | 0.0042021 | NCF2/ITGB2/CYBB | 3 |
| hsa04670 | Leukocyte transendothelial migration | 3/10 | 112/8018 | 0.0002965 | 0.0083481 | 0.0063208 | NCF2/ITGB2/CYBB | 3 |
| hsa04380 | Osteoclast differentiation | 3/10 | 128/8018 | 0.0004394 | 0.0083481 | 0.0063208 | NCF2/CSF1R/BTK | 3 |
| hsa04145 | Phagosome | 3/10 | 152/8018 | 0.0007269 | 0.0103585 | 0.007843 | NCF2/ITGB2/CYBB | 3 |
| hsa04015 | Rap1 signaling pathway | 3/10 | 210/8018 | 0.001855 | 0.0211464 | 0.0160111 | ITGB2/FPR1/CSF1R | 3 |
| hsa05221 | Acute myeloid leukemia | 2/10 | 67/8018 | 0.0029646 | 0.0248549 | 0.018819 | CSF1R/BCL2A1 | 2 |
| hsa04664 | Fc epsilon RI signaling pathway | 2/10 | 68/8018 | 0.0030524 | 0.0248549 | 0.018819 | BTK/ALOX5AP | 2 |
| hsa05150 | Staphylococcus aureus infection | 2/10 | 96/8018 | 0.0059972 | 0.0427299 | 0.0323532 | ITGB2/FPR1 | 2 |
| hsa04064 | NF-kappa B signaling pathway | 2/10 | 104/8018 | 0.0070066 | 0.0437438 | 0.0331209 | BCL2A1/BTK | 2 |
| hsa04066 | HIF-1 signaling pathway | 2/10 | 109/8018 | 0.0076743 | 0.0437438 | 0.0331209 | CYBB/HK2 | 2 |

adj. p-value, adjusted p-value

**Table S7. Summary of patient characteristics**

|  | **Control** | **CRSwNP** | **p-value** |
| --- | --- | --- | --- |
| Total no. of subjects | 24 | 46 |  |
| Sex, male, n (%) | 12 (50%) | 29 (63%) | 0.299 |
| Age (y), mean ± SD | 45.25 ± 3.214 | 47.07 ± 2.437 | 0.6591 |
| History of asthma | 0 (0%) | 17 (36.96%) | 0.0004 |
| History of smoking | 2 (8.3%) | 4 (8.7%) | 0.9597 |

| **Table S8. Primers for RT-qPCR**   \| **Gene** \| **Forward primer** \| **Reverse primer** \| \| --- \| --- \| --- \| \| ALOX5AP \| GATGCGTACCCCACTTTCCTC \| GAATATGCCAGCAACGGACAT \| \| BCL2A1 \| GGCTGGCTCAGGACTATCTG \| CCCAGTTAATGATGCCGTCT \| \| NCF2 \| ATGCTAATAACCAGACAACAG \| GCCATTCTTCATTCACCTT \| \| HCK \| TCGGAGGCAATACATTCT \| GCTGTTGTGGCTATTAGG \| \| BTK \| CTAAGGAAGCGGTGGATT \| TGAGAGCAGCAGAGATAC \| \| CYBB \| ATGCTTGTGGCTGTGATA \| AGACTGACTTGAGAATGGAT \| \| HK3 \| GAGTGACTAGCTTCTCTGTG \| AGCAACTCAGGGTTTCTTC \| \| CD300A \| AAGACCTCAACAATCACAAC \| ACAGAAGCAGCAACAATG \| \| CSF1R \| TTTGGGGCTAGACAGACTGG \| CCTGAGCTGAGTGTGGTCTG \| \| ITGB2 \| GAGCAGTCGTTTGTCATC \| CCAATGTAGCCAGTGTCA \| \| PLEK \| GCTGGTATCCAACCAGTCTG \| CATTGAGCAGCGATGAAGCA \| \| FPR1 \| GCTGTATCTGCTGGCTAT \| GTAACTGATGGTGGTGACT \| \| GAPDH \| GGAGTCAACGGATTTGGTC \| TGGGTGGAATCATATTGGAACAT \| |
| --- | --- | --- | --- | --- | --- | --- | --- | --- | --- | --- | --- | --- | --- | --- | --- | --- | --- | --- | --- | --- | --- | --- | --- | --- | --- | --- | --- | --- | --- | --- | --- | --- | --- | --- | --- | --- | --- | --- | --- | --- | --- | --- |

**Table S9. ROC curve analysis of the individual hub genes**

| Gene | AUC | Std. error | 95% CI | p-value |
| --- | --- | --- | --- | --- |
| ALOX5AP | 0.7698 | 0.04056 | 0.6903 to 0.8493 | <0.0001 |
| BCL2A1 | 0.7639 | 0.05754 | 0.6511 to 0.8767 | 0.0003 |
| BTK | 0.7029 | 0.0706 | 0.5645 to 0.8413 | 0.0108 |
| NCF2 | 0.8913 | 0.04068 | 0.8116 to 0.971 | <0.0001 |
| HCK | 0.8185 | 0.05195 | 0.7167 to 0.9203 | <0.0001 |
| HK3 | 0.7136 | 0.06324 | 0.5897 to 0.8376 | 0.004 |
| CYBB | 0.8418 | 0.0648 | 0.7148 to 0.9688 | <0.0001 |
| 7 gene combination | 0.9354 | 0.03008 | 0.8764 to 0.9944 | <0.0001 |

AUC, area under curve

CI, confidence interval

**Table S10. Summary of patient characteristics of the four datasets**

|  | Control | CRSwNP |
| --- | --- | --- |
| GSE136825 |  |  |
| Total no. of subjects | 28 | 42 |
| Sex, Male, n(%) | not mentioned | not mentioned |
| Age(y), mean ± SD | not mentioned | not mentioned |
| Allergic rhinitis in history | 0 (0%) | 0 (0%) |
| Asthma in history | 0 (0%) | 0 (0%) |
| acute infections | 0 (0%) | 0 (0%) |
| used intranasal or systemic corticosteroids or antibiotics within 4 weeks before the surgery | 0 (0%) | 0 (0%) |
| ASA-intolerance | 0 (0%) | 0 (0%) |
| Smoke in history | not mentioned | not mentioned |
| GSE36830 |  |  |
| Total no. of subjects | 6 | 6 |
| Sex, Male, n(%) | 2 (33%) | 4 (67%) |
| Age(y), mean ± SD | 36 ± 6 | 38 ± 5 |
| Allergic rhinitis in history | 0 (0%) | 1 (17%) |
| Asthma in history | 0 (0%) | 1 (17%) |
| acute infections | not mentioned | not mentioned |
| used intranasal or systemic corticosteroids or antibiotics within 4 weeks before the surgery | 0 (0%) | 1 (17%) |
| ASA-intolerance | not mentioned | not mentioned |
| Smoke in history | not mentioned | not mentioned |
| GSE23552 |  |  |
| Total no. of subjects | 17 | 11 |
| Sex, Male, n(%) | 9 (53%) | 5 (45%) |
| Age(y), mean ± SD | 31.22 ±2.913 | 40 ±2.788 |
| Allergic rhinitis in history | 4 (24%) | 1 (17%) |
| Asthma in history | 0 (0%) | 1 (17%) |
| acute infections | 0 (0%) | 0 (0%) |
| used intranasal or systemic corticosteroids or antibiotics within 4 weeks before the surgery | not mentioned | not mentioned |
| ASA-intolerance | 0 (0%) | 5 (45%) |
| Smoke in history | 0 (0%) | 0 (0%) |
| GSE72713 |  |  |
| Total no. of subjects | 3 | 6 |
| Sex, Male, n(%) | 1 (67%) | 4 (67%) |
| Age(y), mean ± SD | 48.7±7.6 | 46.8±4.8 |
| Allergic rhinitis in history | 0 (0%) | 1 (17%) |
| Asthma in history | 0 (0%) | 1 (17%) |
| acute infections | not mentioned | not mentioned |
| used intranasal or systemic corticosteroids or antibiotics within 4 weeks before the surgery | 0 (0%) | 0 (0%) |
| ASA-intolerance | not mentioned | not mentioned |
| Smoke in history | not mentioned | not mentioned |
